# Prediction of axial capacity of corrosion-affected RC columns strengthened with inclusive FRP

**DOI:** 10.1038/s41598-024-64756-4

**Published:** 2024-06-18

**Authors:** Prashant Kumar, Harish Chandra Arora, Aman Kumar, Dorin Radu

**Affiliations:** 1 Academy of Scientific and Innovative Research (AcSIR), Ghaziabad, 201002 India; 2 Structural Engineering Department, CSIR—Central Building Research Institute, Roorkee, Uttarakhand 247667 India; 3 Faculty of Civil Engineering, Transilvania University of Brașov, 500152 Brașov, Romania

**Keywords:** RC column, FRP, Corrosion, Machine learning, XGBoost, Strengthening, Civil engineering, Computational science

## Abstract

The primary cause behind the degradation of reinforced concrete (RC) structures is the propagation of corrosion in the steel-RC structures. Nowadays, numerous retrofitting techniques are available in the construction sector. Fiber-reinforced polymer (FRP) is one of the efficient rehabilitation measures that can be implemented on corroded structures to enhance structural capacities. However, the estimation of axial strength of FRP-strengthened columns affected by corrosion has been a challenging and tedious task in the laboratory as well as on the site. Considering such shortcomings, the prediction of axial capacity can be done using various analytical methods and artificial intelligence (AI) techniques. In this study, a comprehensive dataset of circular columns was extracted from the literature to predict the axial strength of FRP-wrapped and unstrengthened RC corroded columns. The laboratory results from the assembled dataset were compared to corresponding values estimated using relevant design codes provided by American Concrete Institute (ACI 440.2R-17 and ACI 318-19), and Bureau of Indian Standard (IS 456:2000). Five machine learning models were employed on columns to predict the axial load carrying capacity of FRP-strengthened and un-strengthened RC corroded columns. The results discovered that the extreme gradient boosting (XGBoost) model achieves superior accuracy with the least errors and could be used by the scientific community and FRP applicators to forecast the axial performance of corroded columns strengthened with and without FRP. The findings from the design codes revealed that prediction errors were available in high margins. Furthermore, feature importance analysis was conducted using the Shapley Additive exPlanation algorithm to know the contribution and influence of each input parameter on axial capacity. The feature analysis found that unconfined compressive strength of concrete plays an important role in deciding the axial capacity of columns. Moreover, to enhance the precision of axial capacity computation and improving the overall efficacy in engineering practice, a web-based user-friendly interface was developed for FRP applicators and engineers to simplify the process.

## Introduction

The detrimental corrosive environment adversely affects the structural stability of reinforced concrete (RC) buildings. The structure deteriorates due to long-term exposure in marine coastal areas through the induction of corrosion in the buildings or other exposed RC structures, which causes a reduction in the load-carrying capacity as well as the safety of the structure. Concrete generally renders an alkaline environment to the steel reinforcement. During the hydration of cement, the pore solution achieves a high pH of 12.5 and forms a passive layer surrounding the steel when it interacts with an alkaline solution. A passive coating is a dense and impermeable film of oxide/hydroxide representing a low oxidation rate. However, corrosion begins in the reinforcing steel once the passive layer becomes unstable. Carbonation and chloride-induced corrosion are the two significant phenomena accountable for corrosion in RC elements^1^. These mechanisms are very unusual phenomena that do not attack the concrete instead they attack the steel reinforcement. These are not like sulphate attack that destroys concrete integrity before steel degrades^2^.

Carbonation is a major concern in RC buildings. The atmospheric carbon dioxide (CO_2_) penetrates inside the concrete through microcracks which react with alkaline hydroxides. The CO_2_ gas interacts with water to form carbonic acid which assists in balancing the pH of the pore matrix. Eventually, all the hydroxides get consumed allowing carbonic acid to precipitate which allows pH to fall below 12^2^. As a result, the barrier of the passive layer becomes weak and unstable resulting in rebar corrosion. RC structures are more prone to chloride-contaminated corrosion due to the ingress of chlorides from various sources. Chlorides are induced into the concrete either at the time of construction or can be diffused externally from a salty environment. During the construction stage of concrete specimens, chlorides may be introduced by using accelerators like calcium chloride, by mixing seawater, or by utilizing aggregates dredged from coastal areas. On the other hand, chlorides may be induced from marine coastal surroundings and de-icing salts.

With regard to corrosion, prior research largely emphasizes on causes, mechanisms, and influence of corrosion in RC columns^3^. Vu et al.^4^ discovered that when RC columns were subjected to severe corrosion (20–30%), their lateral load resistance and drift capacity decreased. Jia et al.^5^ recently investigated the impact of seismic activity on bridge columns and discovered that even with 6% corrosion, there was still a significant reduction in ductility displacement. Rajput et al.^6^ emphasized the seismic performance of corroded RC columns and observed a reduction in lateral capacity by 37.1%. Ma et al.^7^ also checked the lateral performance of degraded RC columns and discovered that corroded columns suffer the worst seismic impact when the corrosion loss ratio was 10–20%. Moreover, Meda et al.^8^ found a 30% reduction in the overall capacity of RC columns when subjected to lateral loading.

Modern society now recognizes the importance of upgrading existing infrastructure from a sustainability point of view. The RC structures need to be strengthened rather than demolished by keeping the economy’s financial problems in mind. Several conventional strengthening methods have already been implemented on existing buildings like steel and concrete jacketing, which ultimately enhances the load-bearing strength and ductility of the structures. However, above said methods become obsolete and turned out to be the costliest and carry certain disadvantages. Concrete jacketing results in enlarged areas by casting additional concrete over the entire portion of the column, whereas steel jacketing is more susceptible to internal corrosion, incorporates antirust work, and has handling issues ^9^. Fiber-reinforced polymer (FRP) is a composite material that addresses the shortcomings of traditional retrofitting methods and exhibits several advantages namely; superior capacity, low weight, corrosion resistance, high endurance, and easy usage in the field ^10^. Owing to their structural advantages such as resistance to corrosion, FRP has been employed in a diverse range of structural elements such as beam, column, beam-column joint, slab, etc. However, certain limitations that limit the use of FRPs in the strengthening structure are incompatibility at elevated temperatures, poor breathability, and permeability issues with the concrete as well as masonry surface.

**Figure 1:**
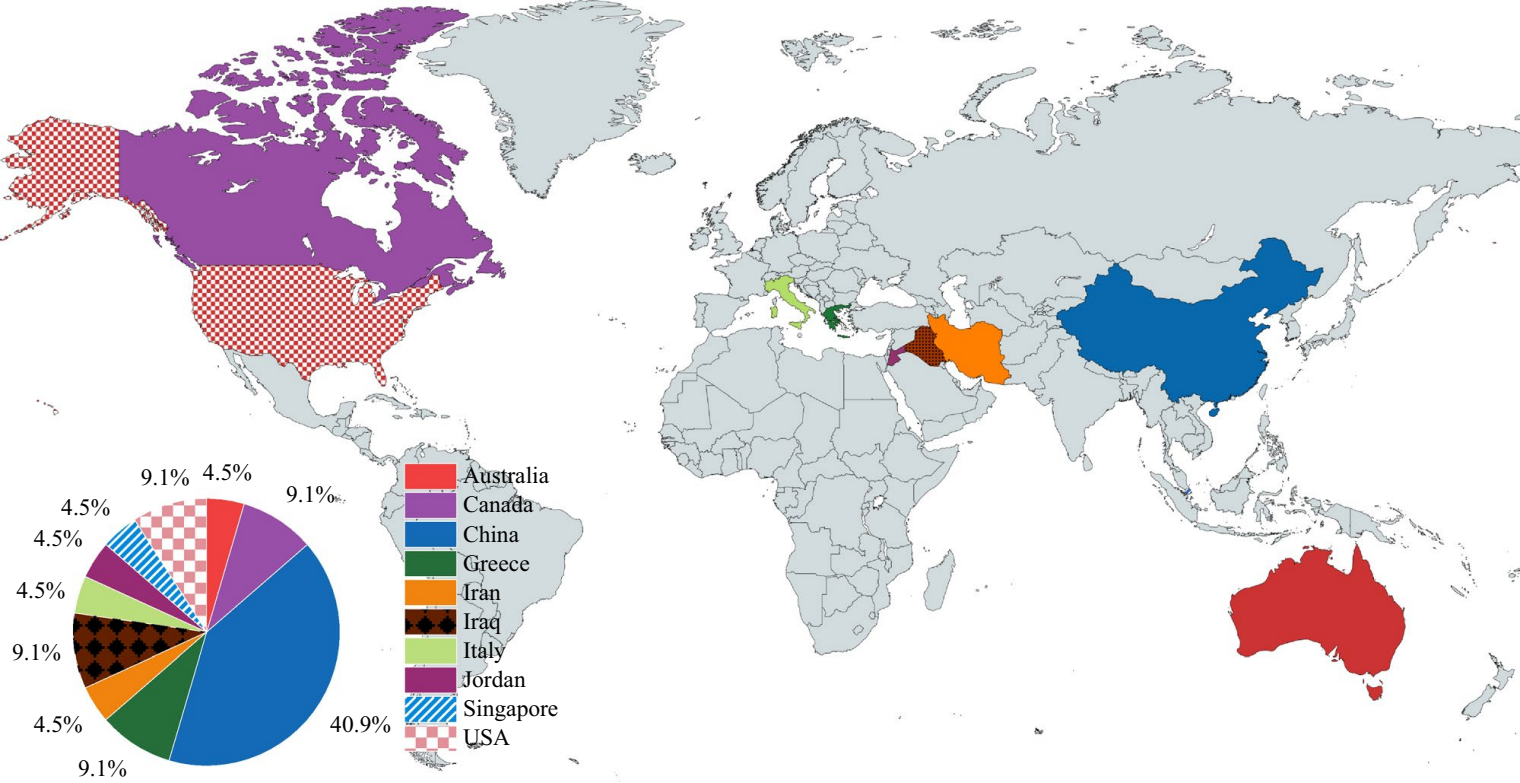
Contribution of the different countries in the experimental dataset.

Several research investigations on damaged/corroded RC columns strengthened with FRP have been done in the published literature. Al-Akhras and Al-Mashraqi^11^ applied a hybrid repair method of carbon FRP (CFRP) and near surface-mounted strips around corroded columns. The test findings demonstrated that, with an average load rise of 16%, the hybrid method of repair considerably restores the nominal axial load-bearing capacity with lower deformability and stiffness. Additionally, CFRP’s volumetric ratio was observed as the key reason behind the capacity enhancement of RC columns affected by corrosion as studied by Chotickai et al.^12^. Kalyoncuoglu et al.^13^ noticed the confining effect of CFRP sheets on substandard corroded RC columns made with a low-quality grade of concrete. The test results showed that the capacity and ductility of damaged columns were greatly improved. Moreover, Yingwu et al.^14^ concluded from their findings that CFRP-wrapped damaged RC columns were more efficient than newly built CFRP-wrapped columns. In this study, the dataset was collected from different countries as shown in Fig. 1.

Axial capacity is the major engineering property used for designing columns. For estimating the axial strength capacity of corroded columns strengthened and un-strengthened with FRP composites, scarce empirical models are available in the global structural engineering community. Due to material uncertainties, complex principles, and various governing factors, precise evaluation or prediction of intricate problems is sometimes not accomplished in many engineering issues^15^. Using the most recent advances in artificial intelligence (AI) or machine learning (ML), a robust and reliable method could be developed to predict the compression capacity of FRP-wrapped and un-wrapped RC corroded columns. The ML technique substantially decreases modelling complexities and diminishes estimating time. Therefore, an ML prediction model for examining the uniaxial load-bearing capacity of RC corroded columns would be quite useful. Artificial neural network (ANN), random forest (RF), support vector machines (SVM), extreme gradient boosting (XGBoost), adaptive neuro-fuzzy inference systems (ANFIS), etc., are the commonly applied ML methods in structural engineering to solve the complex problems.

**Figure 2:**
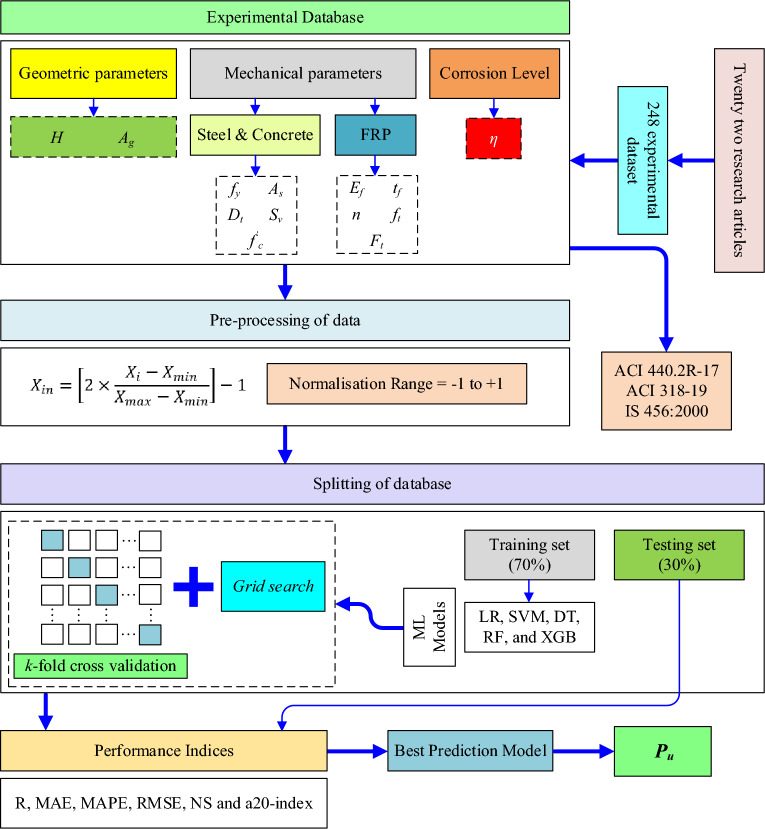
Work methodology of the present work.

Thus, this study aims to predict the axial load-bearing capacity of corroded RC columns strengthened with FRP and without FRP using ML algorithms. The adopted methodology in this work is shown in Fig. 2.

### Application of ML algorithms in columns

In recent years, numerous ML algorithms have been implemented on RC columns to predict the axial capacity. The applications of soft computing methods on RC columns to forecast the axial compression capacity are summarized below:

Cakiroglu et al.^16^ collected a database of 117 test specimens to predict the axial strength capacity of FRP-strengthened RC columns. The used ML methods were kernel ridge regression (KRR), lasso regression, SVM, categorical boosting (CatBoost), adaptive boosting (AdaBoost), gradient boosting machine (GBM), RF, and XGBoost. The predicted findings revealed that the XGBoost algorithm attained a greater precision of 0.9889. Similarly, Cakiroglu et al.^17^ applied linear regression (LR), RF, AdaBoost, GBM, Light GBM, XGBoost, and CatBoost algorithms on concrete-filled steel tubular (CFST) columns to predict their axial bearing capacity. The results revealed that Light GBM and CatBoost models were more precise with 97.9 and 98.3% accuracy, respectively. Another study on the CFST column was investigated by Wang et al.^18^ to predict the axial load-carrying capacity. The authors considered circular CFST columns and implemented an ensemble method for the prediction. The results show that the suggested ensemble technique outperforms conventional support vector regression (SVR) and RF models.

Karimipour et al.^19^ predicted the load-bearing capacity of Glass-FRP (GFRP) columns by employing multilayer perceptron (MLP), radial basis function (RBF), and SVR methods. The findings demonstrated that MLP, RBF, and SVR models performed with an accuracy of 0.92, 0.93, and 0.92, sequentially. Bakouregui et al.^20^ used the XGBoost technique on FRP-RC columns to forecast the axial bearing capacity. For this study, 283 specimens were amassed from the published experimental studies. The study demonstrated that the XGBoost model outperforms all analytical methods with high accuracy and fewer errors. Arora et al.^21^ predicted the load-bearing strength of FRP-wrapped RC columns by implementing the ANN algorithm and fourteen analytical models. It was observed that the ANN model achieved an accuracy of 0.9758 and outperformed ML as well as analytical models.

Raza et al.^22^ employed the ANN model on GFRP-strengthened columns to predict axial loading capacity by considering 279 specimens. The suggested model had an accuracy of 0.9208, indicating strong agreement with both experimental and empirical results. Kumar et al.^23^ predicted the uniaxial load-bearing capacity of RC columns influenced by corrosion using ANN algorithms. The established model predicts axial capacity with an accuracy of 0.9990.

## Research significance

The axial load-carrying capacity of columns has been largely affected due to the presence of corrosion in structural elements. FRP confinement of the RC columns proved to be the best retrofitting technique in the structural engineering community because of its intrinsic corrosion-resistant characteristics. Nevertheless, estimating the axial capacity of FRP-strengthened and unstrengthened corroded RC columns is a laborious task, time-consuming, and required high-cost equipment. Moreover, existing analytical models depend on various assumptions and restricted datasets. Because of the complexity of evaluating the load-carrying capacity of RC corroded columns, conventional methods are not suitable to evaluate the axial performance of corroded RC columns. This study developed ML models (LR, SVM, decision tree (DT), RF, and XGBoost) for predicting the load-bearing capacity of FRP-strengthened and unstrengthened corroded RC columns. These methods have the potential to replace time-consuming techniques and can quickly predict the axial behaviour of corroded columns. This work is the first attempt in the scientific community to develop ML models to predict the axial load-carrying capacity of FRP-strengthened and unstrengthened corroded RC columns. The methodology described in this research is given in a straightforward computational framework, which renders them very accurate and applicable for practical use.

## Methodology of study

### Data collection and description

A database comprising two hundred and forty-eight datasets was obtained from published literature to predict the load-carrying capacity of the corroded RC columns wrapped with and without FRP composite. The sources of data include laboratory studies of corroded RC columns subjected to axial loading. In this article, 102 specimens were FRP-confined corroded RC columns and 146 specimens were unconfined corroded specimens collected from the literature^7,8,11,14,24–41^. The details of the collected dataset can be obtained from Table S1 and Table S2 of the supplementary file. The independent variables chosen for the study include geometrical properties of the cross-section such as the height of the column $\left( H \right)$ and gross area of the cross-section $\left( {A_{g} } \right)$, concrete grade $\left( {f_{c}{\prime} } \right)$, reinforcement characteristics such as yield strength of longitudinal steel bar $\left( {f_{y} } \right)$, area of longitudinal bars $\left( {A_{s} } \right)$, diameter of transverse reinforcement $\left( {D_{t} } \right)$, and stirrup spacing $\left( {S_{v} } \right)$, FRP fabric characteristics such as elastic modulus of FRP $\left( {E_{f} } \right)$, tensile capacity of FRP $\left( {f_{t} } \right)$, number of FRP layers $\left( n \right)$, thickness of FRP composites $\left( {t_{f} } \right)$, and FRP type $\left( {F_{t} } \right)$, and the percentage of corrosion $\left( \eta \right)$. On the other hand, axial capacity $\left( {P_{u} } \right)$ is the dependent variable (output parameter). The summary of a dataset with all parameters is demonstrated in Table 1. In addition, the database statistical summary is illustrated in Table 2. The distribution of the input and output variables is shown in Fig. S1 of the supplementary file.

**Table 1:** Information of the database collected in this study.

References	*H*	*A*_*g*_	$f^{\prime}_{c}$	*f*_*y*_	*A*_*s*_	*D*_*t*_	*S*_*v*_	*E*_*f*_	*f*_*t*_	*n*	*t*_*f*_	*F*_*t*_	*η*	*P*_*u*_
^7^	1000	53,092.91	32.4	373.2	1205.21	8	100	0	0	0	0	0	4.1–15.1	258–1548.1
^8^	1500	90,000	20	520	801	8	300	0	0	0	0	0	11.9–23.38	57.98–98.33
^11^	1000	62,500	37.5	420	256.25	6	6	0	0	0	0	0	9.5–13.3	1320–1339
^15^	300	17,671.46	38.47	363.37	452.39	6	100	240	3400	1	0.167	1	10–35	477–1039
^25^	300	7853.98	33.6	475	169.65	4	55	230	4000	1–2	0.167	1	8.89–28.95	441–742
^26^	320	40,000	30	550	452	5	140	75–235	1500–3500	2–4	0.13–0.17	1–2	5.53–7.92	1510.60–2333.40
^27^	1000	49,087.38	30.5	500	805.03	10	50	24	1500	1	3	2	25–50	1271–2298
^28^	1016	73,061.66	23.5	483	1059.39	5	44	230	3400	2	0.167	1	8.5–9.3	3547–3659
^29^	457–914	18,145.84–32,365.47	21–34	483	212.31–566.40	6.4	25	228	3790	1	0.17	1	20–36	645–1926
^30^	300	17,671.45	29.8	483	314.55	6	100	15.6–44.7	190–342	1–2	0.13–0.17	1–2	15	473.59–1175.15
^31^	400	7853.98	31.5	483	201.06	4	70	75–235	1342.6–3872.2	2	0.13–0.17	1–2	15.44–26.60	209.5–1287
^32^	300	17,671.46	40.5	483	236.80	4	25	21	420	1–2	1.7	2	5.35–15.55	847–1617
^33^	457	18,145.84	21	483	212.31	3.7	25	227	3790	1	0.16	1	20.00	645–774
^34^	300	17,671.46	39.1	527	157.28	6	135	233	3548	1–3	0.13	1	5–10	1191.94–2281.21
^35^	305	18,145.84	50	483	283.08	6	25	75	720	1	1.3	2	10.00	962–1110
^36^	1350	70,685.83	30	404.97	918.92	8	100	0	0	0	0	0	5–22.5	1312.83–1996.6
^37^	300	7853.98	27.93	332.3	169.65	4	55	0	0	0	0	0	10–30	244–331
^38^	1100	70,685.83	30.25	373.3	1604.57	8	87.5	0	0	0	0	0	3.38–8.41	62.16–70.40
^39^	600	62,500	60.6	427.2	800	16	100	0	0	0	0	0	4.4–13.7	2200–3150
^40^	375–2300	34,000–148,000	19.43–41.24	359–525	523.6–3515.4	4–10	30–150	0	0	0	0	0	0.0064–0.5385	156.42–1220.70
^41^	1100–1260	40,000–60,000	9.6–18.42	386–520	618–924	6–8	80–300	0	0	0	0	0	4–20	117.72–222.36
^42^	750	14,400	27.93	333.2	313.92	6	65	0	0	0	0	0	3–17	215–460

**Table 2:** Statistical parametric description of input and output parameter.

Parameters	Symbol	Unit	Min	Max	Average	Skewness
Height of column	$H$	mm	300	2300	751.81	0.8617
Gross area of column	${A}_{g}$	mm^2^	7853.98	148,000	48,735	0.9531
Compressive strength of concrete	${f}_{c}{\prime}$	MPa	9.6	60.6	31.68	1.02
Yield strength of longitudinal steel	${f}_{y}$	MPa	332.3	550.00	447.74	-0.1578
Area of longitudinal bars	${A}_{s}$	mm^2^	157.28	3515.40	803.50	1.64
Diameter of transverse steel	${D}_{t}$	mm	3.7	16	6.73	2.06
Stirrup spacing	${S}_{v}$	mm	6	300	103.53	1.81
Modulus of Elasticity of FRP	${E}_{f}$	GPa	0	240	56.69	1.31
Tensile strength of FRP	${f}_{t}$	MPa	0	4000	902.68	1.28
Nos. of FRP layers	*n*	–	0	4	0.6612	1.43
FRP thickness	${t}_{f}$	mm	0	3	0.1951	3.93
Type of fabric	${F}_{t}$	–	0	2	0.6	0.8397
Corrosion percentage	*η*	%	0.0064	50	10.17	1.33
Axial strength of the column	${P}_{u}$	kN	57.98	3659	826.66	1.33

**Figure 3:**
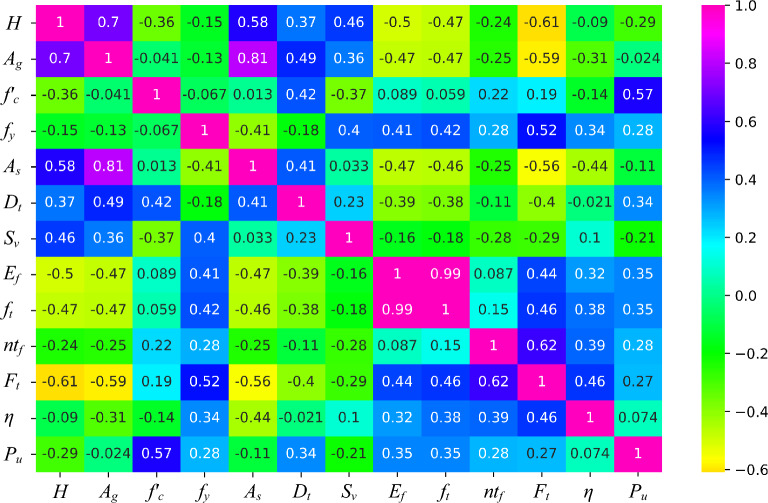
Heat plot.

The relationship between input and output parameter has been discussed in Fig. 3. As mentioned earlier, the features including $H$, $A_{g}$, $f_{c}{\prime}$, $f_{y} , A_{s} , D_{t} , S_{v} , E_{f} , f_{t} , n, t_{f} ,F_{t} ,$ and $\eta$ forms 12 $\times$ 1 matrix, while the target variable $P_{u}$ forms 1 $\times$ 1 matrix. In the broadest sense, correlation pertains to the statistical linear relationship between two variables. A table that lists the correlation coefficients between input variables is known as a correlation matrix. The correlation between two independent variables is discussed in the heat plot as shown in Fig. 3. The plot illustrates that certain variables are highly correlated with one another. The figure depicts a correlation between $P_{u}$ and $f_{c}{\prime}$ with a coefficient of 0.57. The $D_{t}$ attains a correlation coefficient of 0.34 with $P_{u}$. Moreover, correlation observed between $E_{f}$ and $P_{u}$ with a coefficient of 0.34. Additionally, $f_{t}$ had revealed a correlation coefficient with $P_{u}$ of 0.35.

### Processing of data

To predict the axial capacity using ML techniques, it is necessary to split the dataset into two categories i.e., training and testing. In the paper, seventy percent of the data values were considered for training part, whereas testing phase belongs to remaining data values. Data normalization is one of the procedures that have a big impact on the precision of ML model. This is the crucial pre-processing step for the datasets which limits the range of variables to smaller values. The most common data normalization range used in the prediction of models are -1 to 1, 0 to 1, or 0.1 to 0.9, etc.^42^. In this article, the data values were normalized in the range of -1 to 1 using Eq. (1).

1 $$X_{in} = \left[ {2 \times \frac{{X_{i} - X_{min} }}{{X_{max} - X_{min} }}} \right] - 1$$

where $X_{in}$ is the standardized value, $X_{i}$ is the value to be normalized, $X_{max}$ and $X_{min}$ are the maximum and minimum of variable $X_{i}$, respectively.

### Quality assessment

The employed model’s performance was evaluated based on several evaluating criteria such as the linear correlation coefficient (R), the root mean squared error (RMSE), the mean absolute error (MAE), the mean absolute percentage error (MAPE), a20-index, and Nash–Sutcliffe (NS), consecutively. The formulations of the above-mentioned indices are expressed below^43–45^:

2 $$R = { }\frac{{\mathop \sum \nolimits_{{{\text{i}} = 1}}^{{\text{N}}} \left( {y_{e} - \overline{{y_{e} }} } \right)\left( {y_{p} - { }\overline{{y_{p} }} } \right)}}{{\sqrt {\mathop \sum \nolimits_{{{\text{i}} = 1}}^{{\text{N}}} \left( {y_{e} - \overline{{y_{e} }} } \right)^{{2{ }}} \left( {y_{p} - { }\overline{{y_{p} }} } \right)^{2} } }}$$

3 $$MAPE = \frac{1}{N} \mathop \sum \limits_{i = 1}^{N} \left| {\frac{{y_{e} - y_{p} }}{{y_{e} }}} \right| \times 100$$

4 $$RMSE = \sqrt {\frac{{\mathop \sum \nolimits_{i = 1}^{N} \left( {y_{e} - y_{p} } \right)^{2} }}{N}}$$

5 $$MAE = \frac{1}{N} \mathop \sum \limits_{i = 1}^{N} \left| {y_{e} - y_{p} } \right|$$

6 $$NS = 1 - \frac{{\mathop \sum \nolimits_{i = 1}^{N} \left( {y_{e} - y_{p} } \right)^{2} }}{{\mathop \sum \nolimits_{i = 1}^{N} \left( {y_{e} - \overline{{y_{p} }} } \right)^{2} }}$$

7 $$a20 - index = \frac{m20}{N}$$

where $y_{e}$ is the experimental value, $y_{p}$ is the predicted output, $N$ is the number of dataset, and m20 is the ratio of experimental to predicted values that fall in the range of 0.8 and 1.2. The R-value is one of the main evaluating parameters in determining the accuracy of models. The R-value signifies the best fit of the predicted value to the measured value ranging from 0 to 1. The R-value closer to one represents the good bond between experimental and forecasted values. Additionally, lower values of errors including MAPE, RMSE, and MAE indicate higher precision of models. Moreover, MAPE score under 10% indicates that the prediction can be regarded as considerably precise. The MAPE values between 11 and 20%, 21% and 50%, and greater than 51% can be regarded as accurate, decent, and good predictions, respectively^46^.

### Design code provisions

*Model 1* The nominal axial load-bearing capacity of a non-slender concrete member wrapped with an FRP composite system may be estimated as per the requirements of ACI 440.2R-17 guideline^47^.

8 $$\phi P_{n} = k\phi \left[ {0.85f^{\prime}_{cc} \left( {A_{g} - A_{st} } \right) + f_{y} A_{st} } \right]$$

For non-prestressed members with spiral reinforcement, $k$ = 0.85, whereas for members with tie reinforcement, $k$ = 0.80.

where $\phi$ = strength reduction factor, $P_{n}$ = nominal axial strength capacity, $f^{\prime}_{cc}$ = confined compressive strength, $A_{g}$ = gross area of the cross-section, $A_{st}$ = total area of longitudinal reinforcement, and $f_{y}$ = yield strength of steel reinforcement.

*Model 2* According to the ACI 318-19^48^, the design axial strength $\phi P_{n}$ of compression members shall not be regarded to be larger than $\phi P_{n,max}$, determined by:

9 $$\phi P_{n,max} = k\phi \left[ {0.85f^{\prime}_{c} \left( {A_{g} - A_{st} } \right) + f_{y} A_{st} } \right]$$

For non-prestressed members with spiral reinforcement, $k$ = 0.85, whereas for members with tie reinforcement, $k$ = 0.80.

where $P_{n,max}$ = maximum allowable value of *P*_*n*_ and $f^{\prime}_{c}$ = compressive strength of concrete.

*Model 3* The following equation may be used to design the members in accordance with IS 456:2000 code^49^.

10 $$P_{u} = 0.4f_{ck} A_{c} + 0.67f_{y} A_{st}$$

The strength of compression members with helical reinforcement that meet the codal criterion is 1.05 times that of equivalent members with lateral ties.

where $P_{u}$ = axial load on the member, $f_{ck}$ = characteristic strength of the concrete, and $A_{c}$ = concrete area.

All of these equations do not include the corrosion factor that affects the overall axial capacity of the member. Also, ACI 318-19 and IS 456:2000 did not take the contribution of FRP as well as corrosion.

### Adopted ML techniques

#### LR

LR model is a supervised ML technique based on regression analysis (RA). RA is a sort of statistical analysis that is frequently utilized to examine the relationship between a dependent variable and one or multiple predictor variables. To predict the output variable’s future state, the relationship is simulated. The RA consisting of a single input variable is known as single regression analysis (SRA), whereas RA has multiple input variables referred to as multiple regression analysis (MRA). LR models are based on linear equations which can be expressed as shown below:

11 $$E\left( y \right) = \beta _{0} + \beta _{1} \mathcal{x} + \epsilon$$

where $E\left( y \right)$ is the predicted value, $\beta_{0}$ is defined as intercept, $\beta_{1}$ is the coefficient of regression, $\mathcal{x}$ is an input parameter and $\epsilon$ is the noise.

MRA involves utilization of two or more independent variables that examine the output variable. For ‘n’ number of input variables, MRA equation can be expressed as below:

12 $$E\left( y \right) = \beta _{0} + \beta _{1} \mathcal{x}_{1} + \beta _{2} \mathcal{x}_{2} + \ldots \ldots \ldots + \beta _{i} \mathcal{x}_{i} + \ldots \ldots \ldots + \beta _{k} \mathcal{x}_{k} + \in$$

where, ${\mathcal{x}}_{i}$ is the $i{\text{th}}$ independent variable, ${\beta }_{i}$ is the coefficient of ${\mathcal{x}}_{i}$, $k$ is number of independent variables, and $\epsilon$ is the noise.

#### SVM

Vapnik et al.^50^ developed the SVM, a supervised ML model, to address multi-dimensional problems. This is a prominent ML technique rooted in statistical learning principles. This method can be applied to solve classification and regression-based problems^51,52^. The method aims to minimize generalized error and is rooted in the principle of structural risk minimization. The technique has plenty of applications in the fields of ML, data mining, function approximation, pattern recognition, etc. An SVM model forms a separating hyperplane that distinguishes between two or more classes. A collection of training samples that represent points in space are mapped to a multidimensional feature space using a hyperplane. SVM has numerous advantages, including the ability to “handle complex space data,” “situations with a wide variety of features than sample count”, “memory efficiency”, and the adaptability to model the decision function using a range of kernel functions.

13 $$z = \mathop \sum \limits_{i = 1}^{m} \left[ {\gamma_{i} \times k\left( {A_{i} , B} \right)} \right] + b$$

In the above equation, $z$ is the target variable, $m$ is the number of support vectors, $A_{i}$ and $\gamma_{i}$ are the $i{\text{th}}$ support vector and its weight, respectively, $B$ is the input sample vector, and $k\left( {A_{i} , B} \right)$ is the kernel function.

#### DT

DT also referred to as classification and regression tree based on the decision-making process. Due to its simplicity in handling the data, it has emerged as one of the most popular algorithms. This method is the supervised ML technique which has numerous applications in classification as well as regression problems. The dataset is divided into subsets according to the input parameters to produce homogeneous subsets with regard to the output variable. A DT model is composed of four components i.e., root node, multi branches, decision nodes, and leaf nodes. The root node represents the first most node of the tree signifying complete dataset. At the branch’s tip, the leaf node is located which denotes a decision that must be taken, while the decision node denotes the condition leading to the partitioning of a dataset, is positioned along the branch.

A tree is created by dividing the initial root node into compact subsets. The entropy, Gini index, information gain, and mean square error (regression problem) are just a few examples of different metrics on which splitting conditions are based. Due to the explicit and logical portrayal of the decision-making process, one of the benefits of DT is its readability and comprehension. They might, however, be prone to overfitting if regularisation is delayed.

#### RF

In 2001, Breiman introduced the RF algorithm, a non-parametric ML technique for model prediction^53^. RF is the popular algorithm used for multiple classification and regression problems. A set of DTs that are frequently used as a standard model were created using various techniques by RF. The structure of the RF algorithm allows for the combination of different DTs rather than only selection based on a single DT. The learning process involves establishing a collection of DTs, each governed by a ‘Bootstrap’ subset, from the initial learning. Multiple DTs that are randomly created and trained with numerous subsets containing various samples can be learned simultaneously using the RF approach. Following the tree principle and random characteristics, a subset of trees in the forest are trained by using randomly dispersed data.

RF has many advantages that make it a suitable ML technique for the prediction of target variable. First, RF is capable of handling several input variables with deficiencies in a small sample length, preventing over-fitting of the model^54,55^. Second, due to the non-parametric nature of the algorithm, no assumptions are required in deciding the correlation between input and target variables^56^. Third, RF is more noise-resistant than a single DT and has no instability difficulties. Its algorithm averages and sums the outcomes from several DTs^53,57^. Fourth, provided certain requirements are met, RF can handle the multicollinearity problem with input variables ^58^.

#### XGBoost

XGBoost is an extended method of gradient boosting method which was developed by Friedman^59^. It is a well-known ML technique recognized for its computational efficiency, mobility, and versatility to model structured databases. It’s a widely utilized ML end-to-end tree-boosting system that can be easily scaled, and favoured by data scientists in the discipline of data analysis to meet many ML challenges^60^. The foundation of XGBoost is the “boosting” idea, which merges the prediction of weak learners with additive training techniques to produce a robust learner. Additionally, this method helps to avoid overfitting problems and strengthens analytical ability. The results are predicted using additive functions expressed below^61^:

14 $$f_{i}^{{\left( p \right)}} = \sum\limits_{{k = 1}}^{p} {f_{k} \left( {\mathcal{x}_{i} } \right)} = f_{i}^{{\left( {p - 1} \right)}} + f_{p} \left( {\mathcal{x}_{i} } \right)$$

In the above equation, $f_{p} \left( {mathcal{x}_{i} } \right)$ referred the learner at step $p$, $f_{i}^{\left( p \right)}$ denotes the prediction at $p$, $f_{i}^{{\left( {p - 1} \right)}}$ referred to the prediction at $\left( {p - 1} \right)$, and $\mathcal{x}_{i}$ denotes input variables.

To reduce the analytical speed and overfitting of the model, the analytical formulation was developed by XGBoost as shown in Eq. (15).

15 $$Objective^{\left( p \right)} = \mathop \sum \limits_{k = 1}^{n} l\left( {\overline{{y_{i} }} , y_{i} } \right) + \mathop \sum \limits_{k = 1}^{p} \sigma \left( {f_{i} } \right)$$

where $l$ represents the loss function, $n$ denotes the number of samples, and $\sigma$ referred to as the regularization term.

## Results and discussion

This part described the predictive performance of design code provisions and ML algorithms such as LR, SVM, RF, DT, and XGBoost using performance indices such as R, RMSE, MAE, MAPE, NS, and a20-index as described in Quality Assessment sub-section. The best fitting of ML models and empirical relations from design codes is signified through Taylor’s plot and error plot. Henceforth, parametric analysis of input features through Shapley Additive exPlanation (SHAP) is presented.

### Outcomes of design code provisions

**Figure 4:**
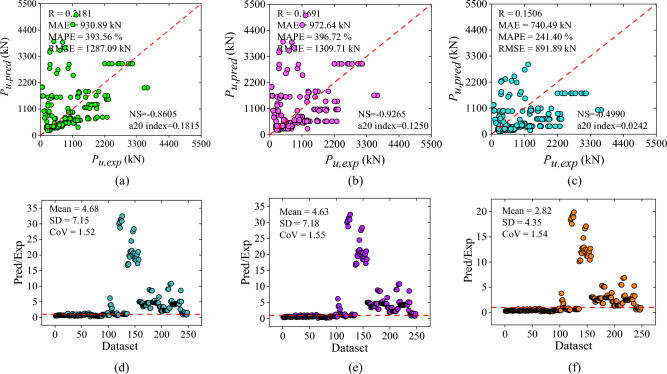
Scatter plot between experimental and predicted axial capacity of design guidelines: (**a**) ACI 440.2R-17, (**b**) ACI 318-19, and (**c**) IS 456:2000; Performance ratio plot of design code provisions: (**d**) ACI 440.2R-17, (**e**) ACI 318-19, and (**f**) IS 456:2000.

The empirical formulations of three design guidelines including ACI 440.2R-17^47^, ACI 318-19^48^, and IS 456:2000^49^ were used to evaluate the load-bearing capacity of corroded RC columns strengthened with and without FRP reinforcement. The result findings show that the correlation coefficient of ACI 440.2R-17^47^, ACI 318-19^48^, and IS 456:2000^49^ guidelines were 0.2181, 0.1691, and 0.1506, sequentially. The prediction of ACI 440.2R-17^47^ resulted in a MAE of 930.89 kN, MAPE of 393.56%, RMSE of 1287.09 kN, NS of -0.8605, and a20-index of 0.1815. Likewise, ACI 318-19^48^ yielded MAE, MAPE, RMSE, NS, and a20-index of 972.64 kN, 396.72%, 1309.71 kN, -0.9265, and 0.1250, respectively. Furthermore, IS 456:2000^49^ yielded MAE, MAPE, RMSE, NS, and a20-index of 740.49 kN, 241.88%, 891.89 kN, − 0.4990, and 0.0242, sequentially. The performance measures of empirical standards can be seen from correlation plots drawn between forecasted and experimental axial capacity i.e., Fig. 4a–c.

The illustration indicates that none of the design formulations had achieved satisfactory accuracy, as evidenced by a significant number of errors. Furthermore, the scatter diagram shows that the dataset was scattered non-uniformly along the best-fit line. Additionally, the scarce cluster of data values was lying on a 45-degree line which represents a significant lack in accuracy of empirical formulations. The ratio of predicted-to-experimental value with respect to the number of dataset plots are also used to demonstrate the findings of the design code provisions, as depicted in Fig. 4d–f. About 8.46, 5.64, and 1.61% of the values had the predicted-to-experimental ratio approximately equal to one for ACI 440.2R-17^47^, ACI 318-19^48^, and IS 456:2000^49^ codal provisions, respectively as presented in Fig. 4d–f.

### Outcomes of ML models

In this article, five ML models (LR, DT, SVM, RF, and XGBoost) were developed for the prediction of axial compressed capacity of RC corroded columns (FRP-wrapped and unstrengthened) using six evaluating measures. The results of the employed ML models have been discussed in the subsequent sections.

#### Performance of LR model

**Figure 5:**
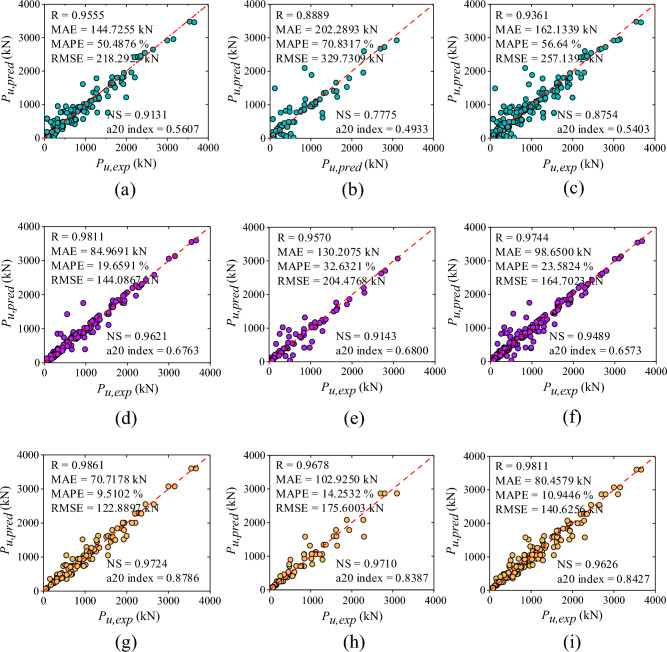

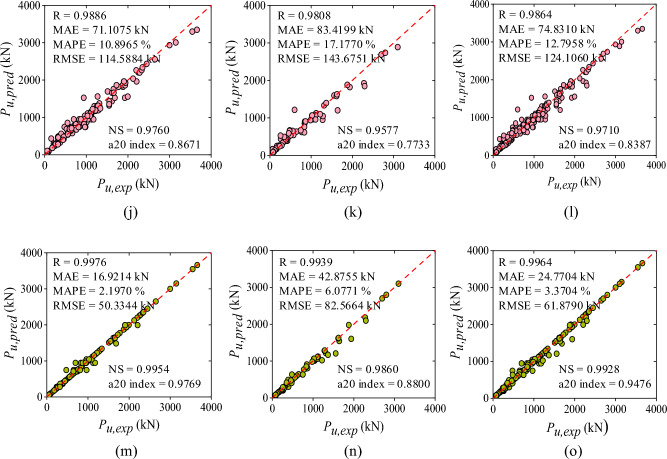
Results of ML models: (**a**) LR- training, (**b**) LR- testing, (**c**) LR- all data, (**d**) SVM- training, (**e**) SVM- testing, (**f**) SVM- all data, (**g**) DT- training, (**h**) DT- testing, and (**i**) DT- all data. (**j**) RF- training, (**k**) RF- testing, (**l**) RF- all data, (**m**) XGBoost- training, (**n**) XGBoost- testing, and (**o**) XGBoost- all data.

**Figure 6:**
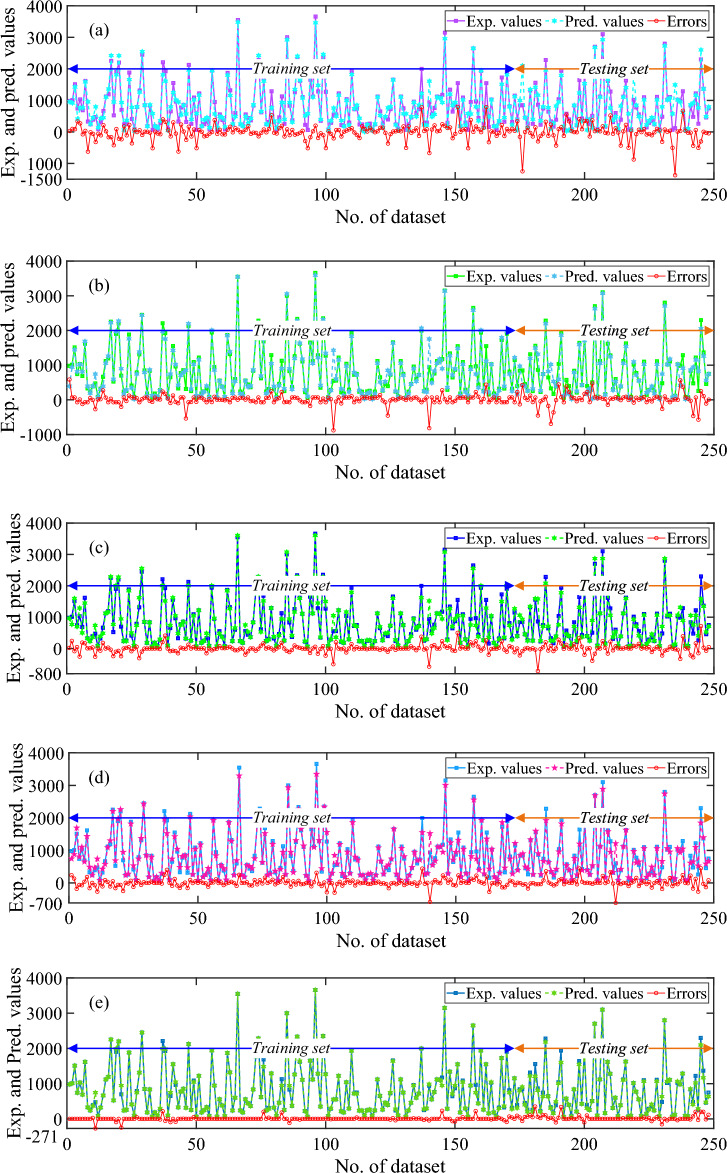
Line plot of the predicted and experimental values with errors (**a**) LR model, (**b**) SVM model, (**c**) DT model, (**d**) RF model, and (**e**) XGBoost model.

**Figure 7:**
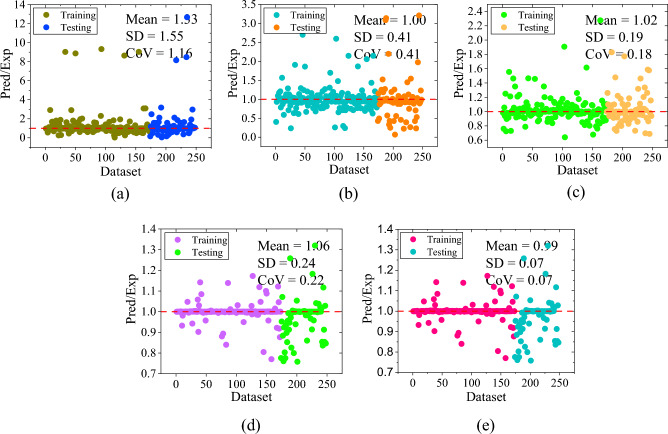
Performance ratio plot: (**a**) LR model, (**b**) SVM model, (**c**) DT model, (**d**) RF model, and (**e**) XGBoost model.

The correlation coefficient (R-value) of LR method for training, testing, and all data set was observed as 0.9555, 0.8889, and 0.9361, sequentially as shown in Fig. 5a–c, respectively. The value of MAE for training, testing, and all data set was observed as 144.72 kN, 202.28 kN, and 162.12 kN, sequentially. The MAPE, RMSE, NS, and a20-index values for the combined dataset were 56.64%, 257.13 kN, 0.8754, and 0.5403, respectively. Correspondingly, for training phase, the values of MAPE, RMSE, NS, and a20-index were noted as 50.48%, 218.29 kN, 0.9121, and 0.5607 respectively whereas, for testing phase, the values were 70.83%, 329.73 kN, 0.7775, and 0.4933, sequentially. The accuracy of LR algorithm was also presented through scatter plots between the actual and predicted values of the axial capacity as shown in Fig. 5a–c. As seen from the plot, the data values were randomly scattered across the fitted line and carried a high range of errors. Moreover, some of the values fall beyond the − 30% to + 30% error range. The line plot of the measured and predicted value with the error distribution is presented in Fig. 6a for the LR model. In training dataset, all the error values were between − 670.58 kN to 792.85 kN whereas, in testing dataset, the range of error was between − 1374.82 kN to 670.27 kN. In training dataset only two values were showed an error range greater than -1000 kN. Furthermore, the performance ratio plot shows that 39.51% of the data points had a predicted-to-experimental value ratio exactly equal to 1.0. as shown in Fig. 7a.

#### Performance of SVM model

This section represents the performance of SVM model using a scatter plot as depicted in Fig. 5d–f**.** As depicted, the R-value of SVM model was observed as 98.11% (training), 95.70% (testing), and 97.44% (all data), sequentially. In terms of MAE, MAPE, and RMSE, MAE = 84.96 kN, 130.20 kN, and 98.65 kN; MAPE = 19.65, 32.63, and 23.58%; RMSE = 144.08 kN, 204.47 kN, and 164.70 kN, using training, testing and entire data set, respectively. Moreover, NS values were 0.9621, 0.9143, and 0.9489 for all three sets whereas, the a20-index was 0.6763, 0.6800, and 0.6573 for all three sets, respectively. The scatter plot shown in Fig. 5d shows a good fit between true and forecasted values in the training phase. In the testing phase as shown in Fig. 5e, the scatter comparisons represent non-uniform scattering of data. Additionally, Fig. 6b depicts the line plot of the SVM model representing measured and predicted values with the distribution of the errors. All the error values in the training dataset lie between − 878.12 kN and 585.47 kN. The error range in the testing dataset was − 690.70 kN to 563.49 kN. Just two values in the training dataset had an error range larger than − 800 kN. In addition, the performance ratio plot represented in Fig. 7b indicates that the predicted-to-experimental ratio for SVM model was equal to one for 49.59% of the data.

#### Performance of DT model

The observed R-values for the DT model were 98.61, 96.78, and 98.11% for the training, testing, and overall dataset, respectively. The R-values demonstrate a good correlation between experimental and predicted capacity. The NS and a20 index values obtained for the training set, testing set, and combined set were as follows: 0.9621 and 0.6763; 0.9143 and 0.6800; and 0.9489 and 0.6573, respectively. In terms of error performance, MAE, MAPE, and RMSE yielded values of 70.71, 102.92, and 80.45 kN; 9.51, 14.25, and 10.94%; and 122.88, 175.60, and 140.92 kN for the training, testing, and entire datasets, respectively. The scatter plot in Fig. 5g–i demonstrates the poor correlation between experimental and predicted axial capacity. It was observed that 19.75% of data values were positioned along the 45-degree line having an error range of − 0.99 and 0.99 kN. Moreover, the majority of data values were located within the error range as shown in the plot. However, some values were still located far from the diagonal line. In addition, Fig. 6c indicates the relationship between measured and predicted values with error distribution in the form of line plot for the DT model. In the training dataset, the error ranged from − 577.66 to 499 kN. However, there was only one instance where the error exceeded − 500 kN. In the testing dataset, the error range extended from − 717 to 717 kN. Furthermore, based on the performance ratio plot as shown in Fig. 7c, 67% of the values had the predicted-to-experimental ratio approximately equal to one for the DT model.

#### Performance of RF model

The RF model demonstrates robust accuracy and precision, evidenced by R-values of 0.9886, 0.9808, and 0.9864 for its training, testing, and overall datasets, respectively. Error measurement indicators, including MAE, MAPE, and RMSE, yielded values of 71.10 kN, 83.41 kN, and 74.83 kN; 10.89%, 17.17%, and 12.79%; and 114.58 kN, 143.67 kN, and 124.10 kN, respectively, for the training, testing, and all datasets. Additionally, NS and a20-index parameters indicate values closer to one i.e., 0.9760, 0.9577, and 0.9710; 0.8671, 0.7733, and 0.8387 for the training, testing, and all data sets, respectively. Figure 5j–l depicts the scatter diagram of the RF model between axial capacity of experimental and predicted values. It was visualized from the plot that very less data values are lying beyond the error range of − 30 to + 30 and 7.22% of data values in case of all dataset are located on the best-fit line within the error range of − 0.97 and 0.75 kN. Similarly, Fig. 6d demonstrate experimental and measured values with error distribution in the form of a line diagram for the RF model. In the training dataset, the error ranged from − 584.92 to 440.79 kN. Notably, only one error value exceeded − 500 kN in this dataset. In the testing dataset, the error range was between − 614.36 and 451.30 kN. Furthermore, the performance ratio values as shown in Fig. 7d were precisely equal to one for 67.33% of the dataset.

#### Performance of XGBoost model

The XGBoost algorithm employed in the study represented the most appropriate results among all other models. The correlation coefficient for the training, testing, and all data sets was 99.76, 99.39, and 99.64%, sequentially, whereas NS and a20-index values were 0.9954, 0.9860, and 0.9928; 0.9769, 0.8800, and 0.9476 for all the three sets, correspondingly. Moreover, the error indices were observed as MAE (16.92, 42.87, and 24.77 kN), MAPE (2.19, 6.07, and 3.37%), and RMSE (50.33, 82.56, and 61.87 kN) for the training, testing, and all data sets, correspondingly. Figure 5m–o depicts the correlation plot between experimental and predicted axial capacity. As seen from the plot, 63.85% of the data values were lying on the diagonal line (best fitting line) having an error range of -0.8 kN and 0.4588 kN which indicates an excellent correlation between actual and forecasted axial capacity of the corroded FRP strengthened and unstrengthened RC column. Moreover, Fig. 6e depicts the line plot of XGBoost model showing experimental and predicted values with error distribution. In the training dataset, the error values ranged from − 270.96 to 229.43 kN, with none falling below − 200 kN. In the testing dataset, the error range was from − 153.68 to 349.97 kN. Furthermore, about 86.69% of the values had the predicted-to-experimental ratio approximately equal to one, as illustrated in Fig. 7e.

### Model’s performance comparison

In this part, the performance of available design codes and established ML models have been compared based on the performance indices parameters, Taylor’s diagram, and violin box plot. When compared to ML methods, it was found that design guidelines outcomes significantly diverge from experimental results. ML techniques outperform all design code outcomes by a substantial margin demonstrating significantly lower values of RMSE, MAE, and MAPE, along with higher values of R, a20-index, and NS. As per performance indices, it was worth mentioning that the XGBoost model was considered the best-performing model because of its excellent precision including lower error values. Specifically, the correlation coefficient (R), a20-index, and NS values of the XGBoost model were 6.44, 75.38, and 13.41% superior compared to those of the LR model, respectively. Additionally, error metrics like RMSE, MAPE, and MAE of the XGBoost model were substantially reduced by 75.93%, 94.05%, and 84.72% in comparison to LR model. When compared with SVM model, the XGBoost model displays superior results. Precisely, its R-value, a20-index, and NS exceed those of the SVM model by 2.25, 44.16, and 4.62% correspondingly. Moreover, RMSE, MAPE, and MAE of XGBoost model were 62.43%, 85.70%, and 74.89% lower when related to SVM model, respectively.

Furthermore, the XGBoost model outperforms the DT model exhibiting higher values for the R-value, NS, and a20-index by 1.55, 3.13, and 12.44%, sequentially. Conversely, statistical errors like RMSE, MAPE, and MAE of XGBoost model were observed 56.09, 69.19, and 69.21% lower than that of the DT model, respectively. In comparison with the RF model, it was noteworthy that the XGBoost model exhibits R-value, NS, and a20-index of 1.01, 2.24, and 11.49% higher in comparison to the RF model, respectively. In contrast to statistical errors, RMSE, MAPE, and MAE values of XGBoost model were 50.14, 76.65, and 66.89% lower when compared to the RF model, considerably.

**Figure 8:**
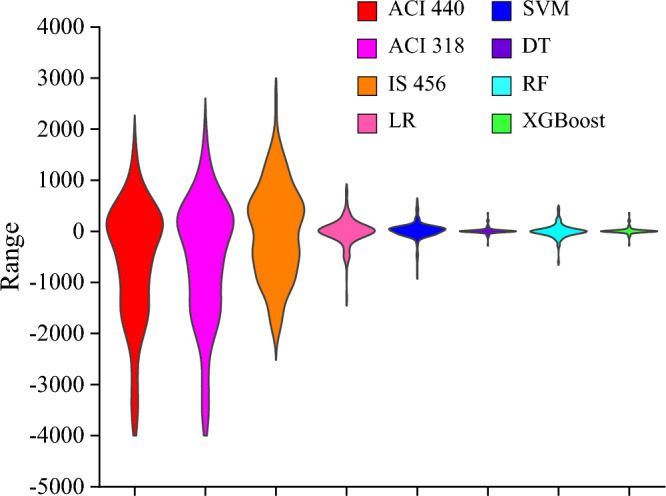
Error distribution using violin plot.

The error distribution between the results of available design guidelines and proposed ML models is shown in the violin plot (Fig. 8). It is visualized from the plot that outcomes obtained from design codes have produced a wide range of errors in comparison to evolved ML models. The range of errors in ACI 440.2R-17^47^, ACI 318-19^48^, and IS 456:2000^49^ were from − 3808.86 to 1672.94 kN, − 3808.80 to 2001.55 kN, and − 1923.18 to 2639.34 kN, sequentially. Conversely, developed ML models had shown a minimum range of errors. As depicted in the plot, the value of error ranges for the LR, SVM, DT, RF, and XGBoost models lying in between − 1374.82 to 792.85 kN, − 878.12 to 585.47 kN, − 717 to 717 kN, − 614.36 to 451.30, and − 270.96 kN to 349.97 kN, respectively. The results indicate that the LR model was the worst-performing model, whereas the XGBoost model implies a lower range of errors, thereby representing the best-performing model according to the violin plot.

**Figure 9:**
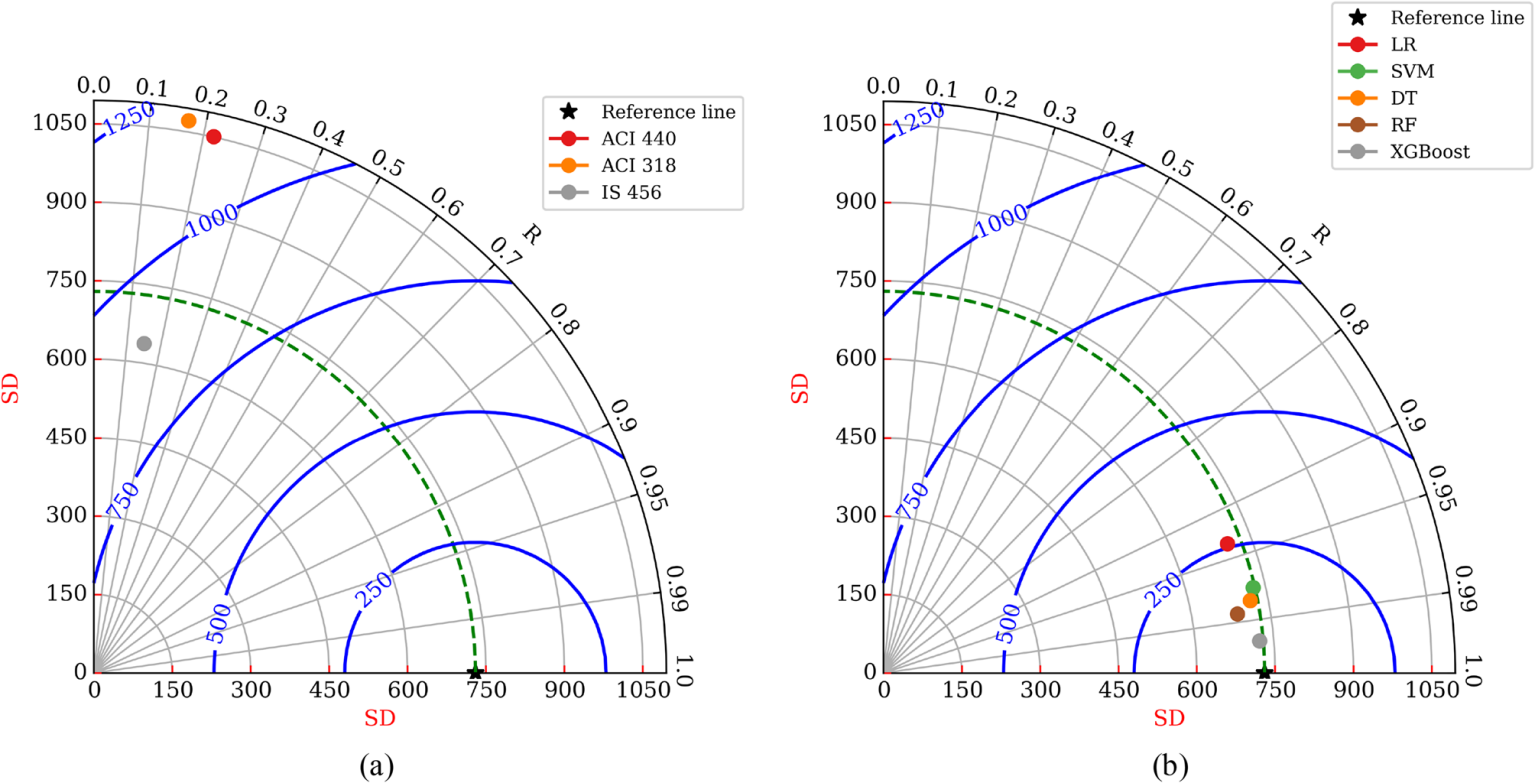
Taylor’s diagram visualization of (**a**) available design guidelines, and (**b**) ML models.

To carry out the comprehensive evaluation of proposed ML models and design standards with in-depth comparison, Taylor’s diagram (Fig. 9) was utilized. The azimuth angle represents the R-value between the experimental and predicted capacity, whereas the radial lines denote the standard deviations for each technique. Furthermore, the RMSE value is signified with concentric circles. In Taylor’s plot, experimental standard deviation arc is represented by a dashed green colour line, and blue colour concentric circles indicate RMSE values. As seen from Fig. 9a, ACI 440.2R-17^47^, ACI 318-19^48^, and IS 456:2000^49^ were located far away from the reference line, whereas developed ML models were lying nearby the reference line (Fig. 9b). It can be witnessed from the graphical representation that standard guidelines results exhibit a higher error range than the ML models. The XGBoost model was indicated by a silver diamond shape whose R-value is 99.76% which was located closer to the horizontal axis, reflecting higher accuracy. Moreover, the standard deviation of XGBoost model (722.84) was closer to that of experimental results (729.92) revealing that XGBoost model exhibits the lowest errors among other models. As a result, it can be concluded that the developed XGBoost model possesses the best prediction ability.

## Feature importance analysis

**Figure 10:**
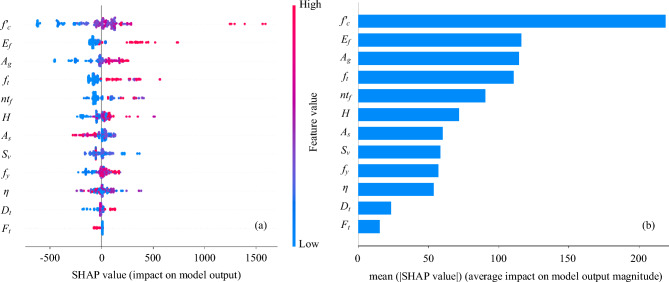
(**a**) SHAP summary plot (**b**) Impact of each contributing feature.

Once the prediction models were optimized and evaluated, the impact and participation of all features on the outcome variable were discussed using Shapley Additive exPlanation (SHAP) values^62^. Figure 10 illustrates the influence of all input variables on the target variable. The first part of Fig. 10 presents a summary plot displaying the SHAP values of all features. The blue colour represents the lesser influence of feature on target axial capacity, whereas the red colour signifies the stronger impact of input variables on the output variable. In addition, second part of Fig. 10 demonstrates the average impact of each input variable on the axial capacity of columns. The attributes were arranged along the y-axis based on their importance in predicting the axial load-bearing capacity of RC columns. The x-axis represents the SHAP values or the average impact on the output of the model. Each dot’s position on the x-axis corresponds to how that feature affected the model’s prediction.

As shown in Fig. 10, the attributes influencing the axial capacity were ordered in decreasing order as follows: concrete grade, modulus of elasticity of FRP, gross area of the cross-section, tensile strength of FRP, percentage of corrosion, thickness of FRP composites, height of the column, yield strength of longitudinal steel bar, stirrup spacing, FRP type, area of longitudinal bars and diameter of transverse reinforcement. Features such as ${f}_{c}{\prime}, {E}_{f},{A}_{g}, {f}_{t}, \eta , n{t}_{f}, H,$ ${\text{and }f}_{y}$ have produced the most significant impact which also satisfies the matrix of correlation coefficients. The Fig. 10b also illustrates that ${f}_{c}{\prime}$ plays a significant role in deciding the axial capacity with a SHAP score of approximately 200 kN. In addition, elastic modulus of FRP $\left({E}_{f}\right)$ is considered the second-best feature in the prediction of axial capacity with a SHAP value of around 125 kN. Moreover, features such as ${S}_{v},$ ${F}_{t},$ ${A}_{s},$ and ${D}_{t}$ are relatively insignificant. Furthermore, the axial capacity is enhanced by reducing transverse reinforcement spacing ${(S}_{v})$, area of longitudinal bar $\left({A}_{s}\right)$ and diameter of stirrups ${(D}_{t}).$

The influence of input variables on the axial capacity as determined in this study aligns with findings from prior literature. Kumar et al.^63^ conducted a sensitivity analysis to explore the effect of input features on the axial compressed capacity of corrosion-affected RC columns, using ANN predictions. Their findings highlighted the significant influence of ${f}_{c}{\prime}$ and ${A}_{g}$ on the column capacity. In another study by Karimipour et al.^19^, the authors predicted the load carrying capacity of GFRP-RC columns using ANN and evolutionary strategy. The impact of input features was prioritized through sensitivity analysis and it was concluded that ${f}_{c}{\prime}$ and cross-sectional width were the most effective parameters.

## Web-based user-friendly interface

**Figure 11:**
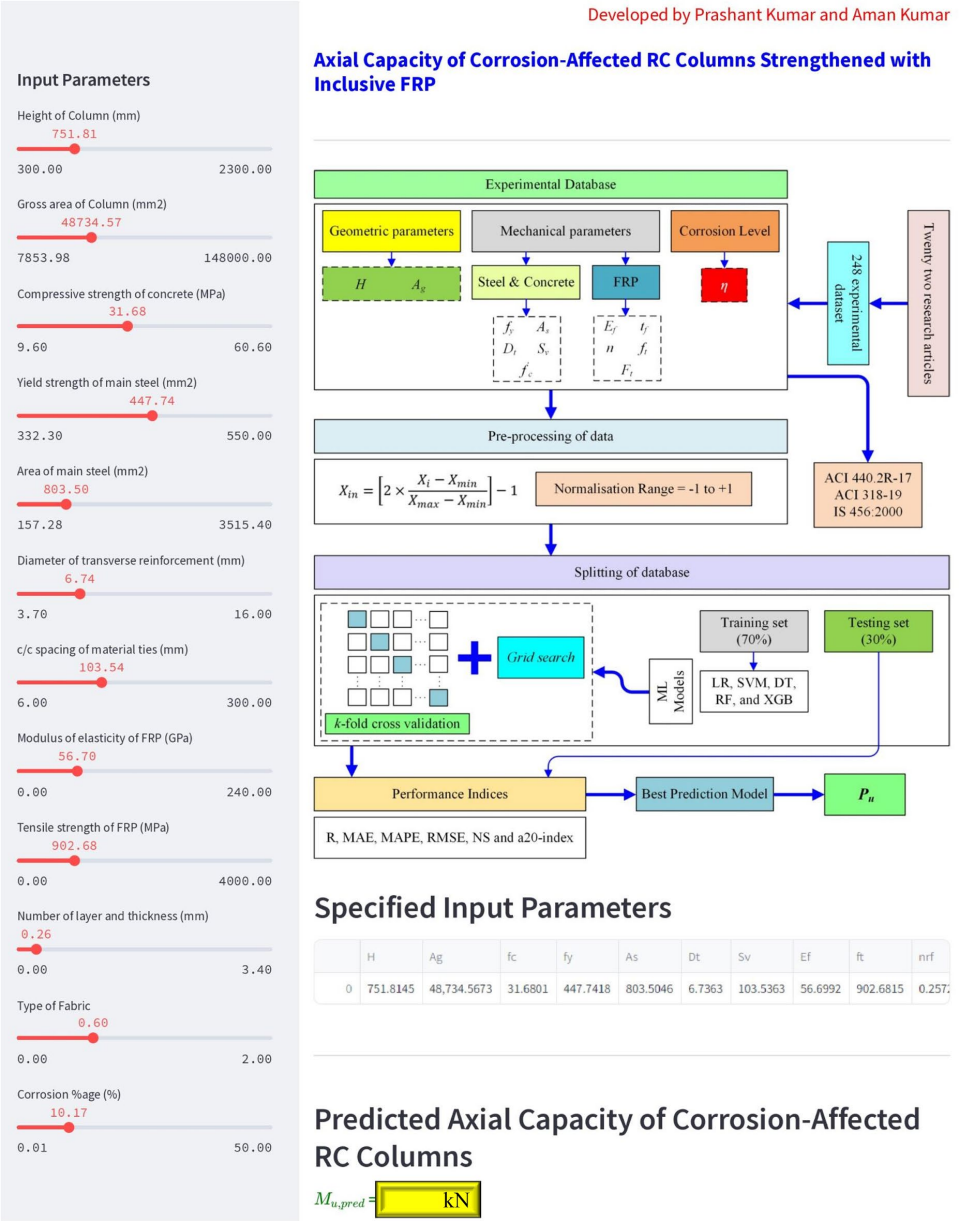
Developed web-based user-friendly tool to predict the axial capacity of corrosion-affected RC Columns strengthened with inclusive FRP.

The developed models used in this study provide a practically applicable ML model for predicting the axial capacity of corrosion-affected RC columns strengthened with inclusive FRP. A real-time, web-based, accessible interface was developed to enable predictions that are accurate, easy to use, and need less computational time. This interface is accessible to all users in the public domain and can be accessed by clicking on the following link.: https://axial-capacity-frp-corrosion.streamlit.app/ . The developed web-based application is shown in Fig. 11.

The web-based tool uses movable sliders to make it easier for users to engage with the web application in order to establish the input parameters ($H$, ${A}_{g}$, ${f}_{c}{\prime}$, ${f}_{y}, {A}_{s}, {D}_{t}, {S}_{v}, {E}_{f}, {f}_{t}, n, {t}_{f},{F}_{t},$ and $\eta )$. The input variables are based on the corresponding ranges that are provided in the collected dataset. On the screen interface, both the user-specified and estimated values are displayed. Significantly, any adjustments to the input parameters quickly compute the target value in a matter of seconds. The online tool can serve as a valuable resource for engineers and FRP applicators by providing more accurate findings without using complex calculations.

## Concluding remarks and promising future recommendations

This paper investigated the efficacy of ML models including LR, DT, SVM, RF, and XGBoost, and formulations extracted from existing design codes such as ACI 440.2R-17^47^, ACI 318-19^48^, and IS 456:2000^49^ to predict the accuracy of axial compressed strength of FRP-strengthened and unstrengthened RC corroded column. A comprehensive literature survey was done to extract the two hundred and forty-eight data values from sources of scientific literature. The collected datasets were divided into two categories i.e., FRP strengthened columns and unstrengthened columns. The features adopted in the study were $H$, ${A}_{g}$, ${f}_{c}{\prime}$, ${f}_{y}, {A}_{s}, {D}_{t}, {S}_{v}, {E}_{f}, {f}_{t}, n, {t}_{f},{F}_{t},$ and $\eta$ whereas ${P}_{u}$ was the outcome variable. Later, feature input analysis and the contribution of all features in forecasting the axial capacity were done using SHAP algorithm.

XGBoost model outperformed all other developed ML models (LR, SVM, DT, and RF). XGBoost model demonstrated an R-value of 0.9976 (training set) and 0.9939 (testing set). XGBoost model also outperformed other developed models in terms of error evaluation. In comparison to LR, SVM, DT, and RF models, XGBoost model exhibited lower error values of 24.77 kN (MAE), 3.37% (MAPE), and 61.87 kN (RMSE). It was observed that employed ML models had achieved R-value in the range of 0.9361 to 0.9964 which was quite exceptional. However, apart from the XGBoost model, all ML models displayed higher error values. The results acquired from empirical relations of design codes illustrate lower performance in comparison to established ML models with a greater margin of accuracy and errors. The R-value of ACI 440.2R-17^47^, ACI 318-19^48^, and IS 456:2000^49^ were observed as 0.2181, 0.1691, and 0.1506, respectively. According to the feature analysis performed by SHAP, ${f}_{c}{\prime}, {E}_{f},{A}_{g}, {f}_{t}, \eta , n{t}_{f}, H, {\text{and }f}_{y}$ have produced a positive impact on the outcome variable. The grade of concrete had shown higher performance contribution on the axial strength of columns in comparison to other features. On the other hand, stirrup spacing, FRP type, diameter of transverse reinforcement, and area of longitudinal bars, had shown inferior performance contribution in forecasting the axial capacity.

### Limitations and uncertainties

The implemented computational ML models can be utilized by the scientific community and research scholars to forecast the axially loaded capacity of corroded RC columns strengthened with and without FRP composites. Nevertheless, the article carries some limitations, even though the XGBoost model has been shown to be a trustworthy and accurate ML model for estimating the axial capacity of columns. The database needs to be updated by including a greater number of experimental studies. Second, the prediction of axial capacity was found to be quite challenging due to the unavailability of analytical models in the literature on the present scope of research.

### Future direction

Some future recommendations are pointed out by the authors for the scientific community; (a) more laboratory studies need to be carried out on corrosion-affected FRP-strengthened columns, (b) analytical models need to be developed in reference to corrosion-affected columns for better prediction of axial capacity, (c) prediction of axial capacity of strengthened and un-strengthened columns including different exposure scenarios, chemical exposure, and aging can be studied, and (d) in future, more advanced ML algorithms can be expected to apply for strength predictions.

### Supplementary Information

Supplementary Information.

## Data Availability

All data generated or analysed during this study are included in this published article (and its supplementary information file).

## List of symbols

$A_{C}$: Area of concrete
$A_{g}$: Gross area of the cross-section
$A_{st} , A_{s}$: Area of longitudinal reinforcement
$D_{t}$: Diameter of transverse reinforcement
$E_{f}$: Modulus of elasticity of FRP
$f_{c}{\prime}$: Grade of concrete
$f^{\prime}_{cc}$: CS of confined concrete
$f_{y}$: Yield strength of non-prestressed steel
$f_{ck}$: Characteristic CS of the concrete
$f_{t}$: Tensile strength of FRP
$$F_{t}$$: FRP type
$H$: Height of the column
$n$: Number of fabric layers
$\eta$: Percentage of corrosion
$P_{u}$, $P_{n}$: Axial load capacity
$P_{n,max}$: Maximum allowable value of *P*_*n*_
$$S_{v}$$: Stirrup spacing
$$t_{f}$$: FRP thickness

## Acronyms

ACI: American concrete institute
AdaBoost: Adaptive boosting
AI: Artificial intelligence
ANFIS: Adaptive neuro-fuzzy inference system
ANN: Artificial neural network
CatBoost: Categorical boosting
CFRP: Carbon-FRP
CFST: Concrete filled steel tubular
CS: Compressive strength
FRP: Fiber -reinforced polymer
GBM: Gradient boosting machine
GFRP: Glass-FRP
IS: Indian standard
KRR: Kernel ridge regression
LR: Linear regression
MAE: Mean absolute error
MAPE: Mean absolute percentage error
ML: Machine learning
MLP: Multilayer perceptron
MRA: Multiple regression analysis
NS: Nash–Sutcliffe
R: Correlation coefficient
RA: Regression analysis
RBF: Radial basis function
RC: Reinforced concrete
RF: Random forest
RMSE: Root mean squared error
SHAP: Shapley additive explanation
SRA: Single regression analysis
SVM: Support vector machines
SVR: Support vector regression
XGBoost: Extreme gradient boosting
