# Supplementary material for: Prediction of axial capacity of corrosion-affected RC columns strengthened with inclusive FRP
Source: Sci Rep. 2024 Jun 18;14:14011. doi: 10.1038/s41598-024-64756-4 (PMC11189465; doi:10.1038/s41598-024-64756-4)
Supplement: Supplementary file 1 — Supplementary Information. [file 41598_2024_64756_MOESM1_ESM.docx]

**Prediction of Axial Capacity of Corrosion-Affected RC Columns Strengthened with Inclusive FRP**

Prashant **Kumar**^1,2^, Harish Chandra **Arora**^1,2^, Aman **Kuma**r^1,2*^, Dorin **Radu**^3^

^1^Academy of Scientific and Innovative Research (AcSIR), Ghaziabad-201002, India

^2^Structural Engineering Department, CSIR—Central Building Research Institute, Roorkee, Uttarakhand 247667, India

^3^Faculty of Civil Engineering, Transilvania University of Brașov, 500152, Romania

*Corresponding author: [aman.cbri21j@acsir.res.in](mailto:aman.cbri21j@acsir.res.in) (Aman Kumar)

**Table S1. Details of the collected dataset.**

| **S. No.** | **Ref.** | ***H*** | ***A_g_*** | ***f'_c_*** | ***f_y_*** | ***A_s_*** | ***D_t_*** | ***S_v_*** | ***E_f_*** | ***f_t_*** | ***n*t_f_*** | ***F_t_*** | ***η (%)*** | ***P_u_*** |
| --- | --- | --- | --- | --- | --- | --- | --- | --- | --- | --- | --- | --- | --- | --- |
| 1 | [1] | 300 | 7853.98 | 33.6 | 475 | 169.65 | 4 | 55 | 230 | 4000 | 0.167 | 1 | 8.89 | 575 |
| 2 |  | 300 | 7853.98 | 33.6 | 475 | 169.65 | 4 | 55 | 230 | 4000 | 0.334 | 1 | 8.89 | 742 |
| 3 |  | 300 | 7853.98 | 33.6 | 475 | 169.65 | 4 | 55 | 230 | 4000 | 0.167 | 1 | 18.16 | 506 |
| 4 |  | 300 | 7853.98 | 33.6 | 475 | 169.65 | 4 | 55 | 230 | 4000 | 0.334 | 1 | 18.16 | 651 |
| 5 |  | 300 | 7853.98 | 33.6 | 475 | 169.65 | 4 | 55 | 230 | 4000 | 0.167 | 1 | 28.95 | 441 |
| 6 |  | 300 | 7853.98 | 33.6 | 475 | 169.65 | 4 | 55 | 230 | 4000 | 0.334 | 1 | 28.95 | 619 |
| 7 | [2] | 320 | 40000 | 30 | 550 | 452.00 | 5 | 140 | 235 | 3500 | 0.26 | 1 | 7.60 | 1651.9 |
| 8 |  | 320 | 40000 | 30 | 550 | 452.00 | 5 | 140 | 235 | 3500 | 0.26 | 1 | 7.48 | 1933.6 |
| 9 |  | 320 | 40000 | 30 | 550 | 452.00 | 5 | 140 | 235 | 3500 | 0.26 | 1 | 6.52 | 1774.3 |
| 10 |  | 320 | 40000 | 30 | 550 | 452.00 | 5 | 140 | 75 | 1500 | 0.34 | 2 | 7.29 | 1510.6 |
| 11 |  | 320 | 40000 | 30 | 550 | 452.00 | 5 | 140 | 75 | 1500 | 0.34 | 2 | 7.61 | 1556.9 |
| 12 |  | 320 | 40000 | 30 | 550 | 452.00 | 5 | 140 | 75 | 1500 | 0.34 | 2 | 7.92 | 1626.9 |
| 13 |  | 320 | 40000 | 30 | 550 | 452.00 | 5 | 140 | 235 | 3500 | 0.52 | 1 | 7.64 | 2253.2 |
| 14 |  | 320 | 40000 | 30 | 550 | 452.00 | 5 | 140 | 235 | 3500 | 0.52 | 1 | 7.86 | 2283 |
| 15 |  | 320 | 40000 | 30 | 550 | 452.00 | 5 | 140 | 235 | 3500 | 0.52 | 1 | 6.12 | 2333.4 |
| 16 |  | 320 | 40000 | 30 | 550 | 452.00 | 5 | 140 | 75 | 1500 | 0.68 | 2 | 6.96 | 1878.7 |
| 17 |  | 320 | 40000 | 30 | 550 | 452.00 | 5 | 140 | 75 | 1500 | 0.68 | 2 | 7.69 | 1725.4 |
| 18 |  | 320 | 40000 | 30 | 550 | 452.00 | 5 | 140 | 235 | 3500 | 0.26 | 1 | 5.53 | 1938.1 |
| 19 |  | 320 | 40000 | 30 | 550 | 452.00 | 5 | 140 | 235 | 3500 | 0.26 | 1 | 5.98 | 1870 |
| 20 | [3] | 1000 | 49087.38 | 30.5 | 500 | 805.03 | 10 | 50 | 24 | 1500 | 3 | 2 | 25.00 | 2298 |
| 21 |  | 1000 | 49087.38 | 30.5 | 500 | 805.03 | 10 | 50 | 24 | 1500 | 3 | 2 | 50.00 | 2208 |
| 22 |  | 1000 | 49087.38 | 30.5 | 500 | 805.03 | 10 | 50 | 24 | 1500 | 3 | 2 | 50.00 | 1905 |
| 23 |  | 1000 | 49087.38 | 30.5 | 500 | 805.03 | 10 | 50 | 24 | 1500 | 3 | 2 | 50.00 | 1902 |
| 24 |  | 1000 | 48400 | 30.5 | 500 | 803.44 | 10 | 50 | 24 | 1500 | 3 | 2 | 50.00 | 1271 |
| 25 | [4] | 1016 | 73061.66 | 23.5 | 483 | 1059.39 | 5 | 44 | 230 | 3400 | 0.334 | 1 | 8.50 | 3659 |
| 26 |  | 1016 | 73061.66 | 23.5 | 483 | 1059.39 | 5 | 44 | 230 | 3400 | 0.334 | 1 | 9.30 | 3547 |
| 27 | [5] | 457 | 18145.84 | 21 | 483 | 212.31 | 6.4 | 25 | 228 | 3790 | 0.17 | 1 | 29.00 | 774 |
| 28 |  | 457 | 18145.84 | 21 | 483 | 212.31 | 6.4 | 25 | 228 | 3790 | 0.17 | 1 | 35.00 | 720.6 |
| 29 |  | 457 | 18145.84 | 21 | 483 | 212.31 | 6.4 | 25 | 228 | 3790 | 0.17 | 1 | 30.00 | 747.3 |
| 30 |  | 457 | 18145.84 | 21 | 483 | 212.31 | 6.4 | 25 | 228 | 3790 | 0.17 | 1 | 36.00 | 645 |
| 31 |  | 914 | 32365.47 | 34 | 483 | 566.40 | 6.4 | 25 | 228 | 3790 | 0.17 | 1 | 20.00 | 1926 |
| 32 |  | 914 | 32365.47 | 34 | 483 | 566.40 | 6.4 | 25 | 228 | 3790 | 0.17 | 1 | 20.00 | 1685 |
| 33 |  | 914 | 32365.47 | 34 | 483 | 566.40 | 6.4 | 25 | 228 | 3790 | 0.17 | 1 | 20.00 | 939 |
| 34 | [6] | 300 | 17671.45 | 29.8 | 483 | 314.55 | 6 | 100 | 15.6 | 190 | 0.17 | 2 | 15.00 | 652.08 |
| 35 |  | 300 | 17671.45 | 29.8 | 483 | 314.55 | 6 | 100 | 44.7 | 342 | 0.13 | 1 | 15.00 | 869.44 |
| 36 |  | 300 | 17671.45 | 29.8 | 483 | 314.55 | 6 | 100 | 15.6 | 190 | 0.17 | 2 | 15.00 | 966.63 |
| 37 |  | 300 | 17671.45 | 29.8 | 483 | 314.55 | 6 | 100 | 44.7 | 342 | 0.13 | 1 | 15.00 | 1175.15 |
| 38 |  | 300 | 17671.45 | 29.8 | 483 | 314.55 | 6 | 100 | 15.6 | 190 | 0.17 | 2 | 15.00 | 743.97 |
| 39 |  | 300 | 17671.45 | 29.8 | 483 | 314.55 | 6 | 100 | 15.6 | 190 | 0.34 | 2 | 15.00 | 837.63 |
| 40 |  | 300 | 17671.45 | 29.8 | 483 | 314.55 | 6 | 100 | 15.6 | 190 | 0.17 | 2 | 15.00 | 480.66 |
| 41 |  | 300 | 17671.45 | 29.8 | 483 | 314.55 | 6 | 100 | 44.7 | 342 | 0.13 | 1 | 15.00 | 830.56 |
| 42 |  | 300 | 17671.45 | 29.8 | 483 | 314.55 | 6 | 100 | 15.6 | 190 | 0.17 | 2 | 15.00 | 749.27 |
| 43 |  | 300 | 17671.45 | 29.8 | 483 | 314.55 | 6 | 100 | 44.7 | 342 | 0.13 | 1 | 15.00 | 1125.67 |
| 44 |  | 300 | 17671.45 | 29.8 | 483 | 314.55 | 6 | 100 | 15.6 | 190 | 0.17 | 2 | 15.00 | 789.91 |
| 45 |  | 300 | 17671.45 | 29.8 | 483 | 314.55 | 6 | 100 | 15.6 | 190 | 0.34 | 2 | 15.00 | 848.23 |
| 46 |  | 300 | 17671.45 | 29.8 | 483 | 314.55 | 6 | 100 | 15.6 | 190 | 0.17 | 2 | 15.00 | 473.59 |
| 47 |  | 300 | 17671.45 | 29.8 | 483 | 314.55 | 6 | 100 | 44.7 | 342 | 0.13 | 1 | 15.00 | 698.02 |
| 48 |  | 300 | 17671.45 | 29.8 | 483 | 314.55 | 6 | 100 | 15.6 | 190 | 0.17 | 2 | 15.00 | 724.53 |
| 49 |  | 300 | 17671.45 | 29.8 | 483 | 314.55 | 6 | 100 | 44.7 | 342 | 0.13 | 1 | 15.00 | 959.56 |
| 50 |  | 300 | 17671.45 | 29.8 | 483 | 314.55 | 6 | 100 | 15.6 | 190 | 0.17 | 2 | 15.00 | 811.12 |
| 51 |  | 300 | 17671.45 | 29.8 | 483 | 314.55 | 6 | 100 | 15.6 | 190 | 0.34 | 2 | 15.00 | 855.30 |
| 52 | [7] | 400 | 7853.98 | 31.5 | 483 | 201.06 | 4 | 70 | 75 | 1342.6 | 0.34 | 2 | 15.44 | 321.6 |
| 53 |  | 400 | 7853.98 | 31.5 | 483 | 201.06 | 4 | 70 | 75 | 1342.6 | 0.34 | 2 | 15.44 | 287.5 |
| 54 |  | 400 | 7853.98 | 31.5 | 483 | 201.06 | 4 | 70 | 75 | 1342.6 | 0.34 | 2 | 15.44 | 280.1 |
| 55 |  | 400 | 7853.98 | 31.5 | 483 | 201.06 | 4 | 70 | 75 | 1342.6 | 0.34 | 2 | 15.44 | 226.3 |
| 56 |  | 400 | 7853.98 | 31.5 | 483 | 201.06 | 4 | 70 | 235 | 3872.2 | 0.26 | 1 | 15.44 | 453.6 |
| 57 |  | 400 | 7853.98 | 31.5 | 483 | 201.06 | 4 | 70 | 235 | 3872.2 | 0.26 | 1 | 15.44 | 390.5 |
| 58 |  | 400 | 7853.98 | 31.5 | 483 | 201.06 | 4 | 70 | 235 | 3872.2 | 0.26 | 1 | 15.44 | 348.3 |
| 59 |  | 400 | 7853.98 | 31.5 | 483 | 201.06 | 4 | 70 | 235 | 3872.2 | 0.26 | 1 | 15.44 | 324.6 |
| 60 |  | 400 | 7853.98 | 31.5 | 483 | 201.06 | 4 | 70 | 75 | 1342.6 | 0.34 | 2 | 15.44 | 287.83 |
| 61 |  | 400 | 7853.98 | 31.5 | 483 | 201.06 | 4 | 70 | 75 | 1342.6 | 0.34 | 2 | 19.24 | 249.5 |
| 62 |  | 400 | 7853.98 | 31.5 | 483 | 201.06 | 4 | 70 | 75 | 1342.6 | 0.34 | 2 | 19.24 | 238.1 |
| 63 |  | 400 | 7853.98 | 31.5 | 483 | 201.06 | 4 | 70 | 75 | 1342.6 | 0.34 | 2 | 19.24 | 209.5 |
| 64 |  | 400 | 7853.98 | 31.5 | 483 | 201.06 | 4 | 70 | 75 | 1342.6 | 0.34 | 2 | 19.24 | 312.33 |
| 65 |  | 400 | 7853.98 | 31.5 | 483 | 201.06 | 4 | 70 | 75 | 1342.6 | 0.34 | 2 | 19.24 | 318.5 |
| 66 |  | 400 | 7853.98 | 31.5 | 483 | 201.06 | 4 | 70 | 235 | 3872.2 | 0.26 | 1 | 26.60 | 1287.0 |
| 67 | [8] | 300 | 17671.46 | 40.5 | 483 | 236.80 | 4 | 25 | 21 | 420 | 3.4 | 2 | 8.75 | 1016 |
| 68 |  | 300 | 17671.46 | 40.5 | 483 | 236.80 | 4 | 25 | 21 | 420 | 3.4 | 2 | 5.35 | 847 |
| 69 |  | 300 | 17671.46 | 40.5 | 483 | 236.80 | 4 | 25 | 21 | 420 | 1.7 | 2 | 13.45 | 1151 |
| 70 |  | 300 | 17671.46 | 40.5 | 483 | 236.80 | 4 | 25 | 21 | 420 | 1.7 | 2 | 12.64 | 864 |
| 71 |  | 300 | 17671.46 | 40.5 | 483 | 236.80 | 4 | 25 | 21 | 420 | 3.4 | 2 | 15.55 | 1283 |
| 72 |  | 300 | 17671.46 | 40.5 | 483 | 236.80 | 4 | 25 | 21 | 420 | 3.4 | 2 | 13.77 | 1617 |
| 73 | [9] | 457 | 18145.84 | 21 | 483 | 212.31 | 3.7 | 25 | 227 | 3790 | 0.16 | 1 | 20.00 | 774 |
| 74 |  | 457 | 18145.84 | 21 | 483 | 212.31 | 3.7 | 25 | 227 | 3790 | 0.16 | 1 | 20.00 | 720.6 |
| 75 |  | 457 | 18145.84 | 21 | 483 | 212.31 | 3.7 | 25 | 227 | 3790 | 0.16 | 1 | 20.00 | 747.3 |
| 76 |  | 457 | 18145.84 | 21 | 483 | 212.31 | 3.7 | 25 | 227 | 3790 | 0.16 | 1 | 20.00 | 645 |
| 77 | [10] | 300 | 17671.46 | 39.1 | 527 | 157.28 | 6 | 135 | 233 | 3548 | 0.13 | 1 | 5.00 | 1367.06 |
| 78 |  | 300 | 17671.46 | 39.1 | 527 | 157.28 | 6 | 135 | 233 | 3548 | 0.13 | 1 | 5.00 | 1191.94 |
| 79 |  | 300 | 17671.46 | 39.1 | 527 | 157.28 | 6 | 135 | 233 | 3548 | 0.26 | 1 | 5.00 | 1616.59 |
| 80 |  | 300 | 17671.46 | 39.1 | 527 | 157.28 | 6 | 135 | 233 | 3548 | 0.26 | 1 | 5.00 | 1882.19 |
| 81 |  | 300 | 17671.46 | 39.1 | 527 | 157.28 | 6 | 135 | 233 | 3548 | 0.39 | 1 | 5.00 | 2281.21 |
| 82 |  | 300 | 17671.46 | 39.1 | 527 | 157.28 | 6 | 135 | 233 | 3548 | 0.39 | 1 | 5.00 | 2124.46 |
| 83 |  | 300 | 17671.46 | 39.1 | 527 | 157.28 | 6 | 135 | 233 | 3548 | 0.13 | 1 | 10.00 | 1314.58 |
| 84 |  | 300 | 17671.46 | 39.1 | 527 | 157.28 | 6 | 135 | 233 | 3548 | 0.13 | 1 | 10.00 | 1260.51 |
| 85 |  | 300 | 17671.46 | 39.1 | 527 | 157.28 | 6 | 135 | 233 | 3548 | 0.26 | 1 | 10.00 | 1658.64 |
| 86 |  | 300 | 17671.46 | 39.1 | 527 | 157.28 | 6 | 135 | 233 | 3548 | 0.26 | 1 | 10.00 | 1618.00 |
| 87 |  | 300 | 17671.46 | 39.1 | 527 | 157.28 | 6 | 135 | 233 | 3548 | 0.39 | 1 | 10.00 | 1943.15 |
| 88 |  | 300 | 17671.46 | 39.1 | 527 | 157.28 | 6 | 135 | 233 | 3548 | 0.39 | 1 | 10.00 | 1938.56 |
| 89 | [11] | 305 | 18145.84 | 50 | 483 | 283.08 | 6 | 25 | 75 | 720 | 1.3 | 2 | 10.00 | 1021 |
| 90 |  | 305 | 18145.84 | 50 | 483 | 283.08 | 6 | 25 | 75 | 720 | 1.3 | 2 | 10.00 | 1090 |
| 91 |  | 305 | 18145.84 | 50 | 483 | 283.08 | 6 | 25 | 75 | 720 | 1.3 | 2 | 10.00 | 1110 |
| 92 |  | 305 | 18145.84 | 50 | 483 | 283.08 | 6 | 25 | 75 | 720 | 1.3 | 2 | 10.00 | 1028 |
| 93 |  | 305 | 18145.84 | 50 | 483 | 283.08 | 6 | 25 | 75 | 720 | 1.3 | 2 | 10.00 | 1090 |
| 94 |  | 305 | 18145.84 | 50 | 483 | 283.08 | 6 | 25 | 75 | 720 | 1.3 | 2 | 10.00 | 1100 |
| 95 |  | 305 | 18145.84 | 50 | 483 | 283.08 | 6 | 25 | 75 | 720 | 1.3 | 2 | 10.00 | 1024 |
| 96 |  | 305 | 18145.84 | 50 | 483 | 283.08 | 6 | 25 | 75 | 720 | 1.3 | 2 | 10.00 | 962 |
| 97 | [12] | 300 | 17671.46 | 38.47 | 363.37 | 452.39 | 6 | 100 | 240 | 3400 | 0.167 | 1 | 10.00 | 841 |
| 98 |  | 300 | 17671.46 | 38.47 | 363.37 | 452.39 | 6 | 100 | 240 | 3400 | 0.167 | 1 | 25.00 | 525 |
| 99 |  | 300 | 17671.46 | 38.47 | 363.37 | 452.39 | 6 | 100 | 240 | 3400 | 0.167 | 1 | 35.00 | 477 |
| 100 |  | 300 | 17671.46 | 38.47 | 363.37 | 452.39 | 6 | 100 | 240 | 3400 | 0.167 | 1 | 10.00 | 1039 |
| 101 |  | 300 | 17671.46 | 38.47 | 363.37 | 452.39 | 6 | 100 | 240 | 3400 | 0.167 | 1 | 25.00 | 1037 |
| 102 |  | 300 | 17671.46 | 38.47 | 363.37 | 452.39 | 6 | 100 | 240 | 3400 | 0.167 | 1 | 35.00 | 1011 |
| 103 | [13] | 1000 | 53092.92 | 32.4 | 373.2 | 1205.21 | 8 | 100 | 0 | 0 | 0 | 0 | 9.5 | 258 |
| 104 |  | 1000 | 53092.92 | 32.4 | 373.2 | 1205.21 | 8 | 100 | 0 | 0 | 0 | 0 | 4.1 | 412 |
| 105 |  | 1000 | 53092.92 | 32.4 | 373.2 | 1205.21 | 8 | 100 | 0 | 0 | 0 | 0 | 9.7 | 395.6 |
| 106 |  | 1000 | 53092.92 | 32.4 | 373.2 | 1205.21 | 8 | 100 | 0 | 0 | 0 | 0 | 9.3 | 688 |
| 107 |  | 1000 | 53092.92 | 32.4 | 373.2 | 1205.21 | 8 | 100 | 0 | 0 | 0 | 0 | 14.7 | 550 |
| 108 |  | 1000 | 53092.92 | 32.4 | 373.2 | 1205.21 | 8 | 100 | 0 | 0 | 0 | 0 | 11.3 | 1032 |
| 109 |  | 1000 | 53092.92 | 32.4 | 373.2 | 1205.21 | 8 | 100 | 0 | 0 | 0 | 0 | 11.5 | 1290 |
| 110 |  | 1000 | 53092.92 | 32.4 | 373.2 | 1205.21 | 8 | 100 | 0 | 0 | 0 | 0 | 13.2 | 1280 |
| 111 |  | 1000 | 53092.92 | 32.4 | 373.2 | 1205.21 | 8 | 100 | 0 | 0 | 0 | 0 | 12.1 | 1548.1 |
| 112 |  | 1000 | 53092.92 | 32.4 | 373.2 | 1205.21 | 8 | 100 | 0 | 0 | 0 | 0 | 15.1 | 1548 |
| 113 | [14] | 1350 | 70685.83 | 30 | 404.97 | 918.92 | 8 | 100 | 0 | 0 | 0 | 0 | 5 | 1996.6 |
| 114 |  | 1350 | 70685.83 | 30 | 404.97 | 918.92 | 8 | 100 | 0 | 0 | 0 | 0 | 12.5 | 1643 |
| 115 |  | 1350 | 70685.83 | 30 | 404.97 | 918.92 | 8 | 100 | 0 | 0 | 0 | 0 | 17.5 | 1550.86 |
| 116 |  | 1350 | 70685.83 | 30 | 404.97 | 918.92 | 8 | 100 | 0 | 0 | 0 | 0 | 22.5 | 1312.83 |
| 117 | [15] | 300 | 7853.982 | 27.93 | 332.3 | 169.65 | 4 | 55 | 0 | 0 | 0 | 0 | 10 | 331 |
| 118 |  | 300 | 7853.982 | 27.93 | 332.3 | 169.65 | 4 | 55 | 0 | 0 | 0 | 0 | 20 | 290 |
| 119 |  | 300 | 7853.982 | 27.93 | 332.3 | 169.65 | 4 | 55 | 0 | 0 | 0 | 0 | 30 | 244 |
| 120 | [16] | 1100 | 70685.83 | 30.25 | 373.3 | 1604.57 | 8 | 87.5 | 0 | 0 | 0 | 0 | 3.38 | 67.385 |
| 121 |  | 1100 | 70685.83 | 30.25 | 373.3 | 1604.57 | 8 | 87.5 | 0 | 0 | 0 | 0 | 3.99 | 66.285 |
| 122 |  | 1100 | 70685.83 | 30.25 | 373.3 | 1604.57 | 8 | 87.5 | 0 | 0 | 0 | 0 | 4.55 | 70.405 |
| 123 |  | 1100 | 70685.83 | 30.25 | 373.3 | 1604.57 | 8 | 87.5 | 0 | 0 | 0 | 0 | 8 | 64.41 |
| 124 |  | 1100 | 70685.83 | 30.25 | 373.3 | 1604.57 | 8 | 87.5 | 0 | 0 | 0 | 0 | 8.41 | 64.45 |
| 125 |  | 1100 | 70685.83 | 30.25 | 373.3 | 1604.57 | 8 | 87.5 | 0 | 0 | 0 | 0 | 7.96 | 65.55 |
| 126 |  | 1100 | 70685.83 | 30.25 | 373.3 | 1604.57 | 8 | 87.5 | 0 | 0 | 0 | 0 | 7.16 | 62.165 |
| 127 | [17] | 600 | 62500 | 60.6 | 427.2 | 800.00 | 16 | 100 | 0 | 0 | 0 | 0 | 4.4 | 3000 |
| 128 |  | 600 | 62500 | 60.6 | 427.2 | 800.00 | 16 | 100 | 0 | 0 | 0 | 0 | 9.4 | 2650 |
| 129 |  | 600 | 62500 | 60.6 | 427.2 | 800.00 | 16 | 100 | 0 | 0 | 0 | 0 | 13.7 | 2200 |
| 130 |  | 600 | 62500 | 60.6 | 427.2 | 800.00 | 16 | 100 | 0 | 0 | 0 | 0 | 4.3 | 3100 |
| 131 |  | 600 | 62500 | 60.6 | 427.2 | 800.00 | 16 | 100 | 0 | 0 | 0 | 0 | 8.9 | 2700 |
| 132 |  | 600 | 62500 | 60.6 | 427.2 | 800.00 | 16 | 100 | 0 | 0 | 0 | 0 | 13 | 2350 |
| 133 |  | 600 | 62500 | 60.6 | 427.2 | 800.00 | 16 | 100 | 0 | 0 | 0 | 0 | 3.7 | 3150 |
| 134 |  | 600 | 62500 | 60.6 | 427.2 | 800.00 | 16 | 100 | 0 | 0 | 0 | 0 | 8 | 2800 |
| 135 |  | 600 | 62500 | 60.6 | 427.2 | 800.00 | 16 | 100 | 0 | 0 | 0 | 0 | 11.3 | 2450 |
| 136 | [18] | 1500 | 90000 | 20 | 520 | 801.00 | 8 | 300 | 0 | 0 | 0 | 0 | 11.9 | 98.33 |
| 137 |  | 1500 | 90000 | 20 | 520 | 801.00 | 8 | 300 | 0 | 0 | 0 | 0 | 12.9 | 95.45 |
| 138 |  | 1500 | 90000 | 20 | 520 | 801.00 | 8 | 300 | 0 | 0 | 0 | 0 | 12.6 | 96.31 |
| 139 |  | 1500 | 90000 | 20 | 520 | 801.00 | 8 | 300 | 0 | 0 | 0 | 0 | 21.2 | 83.26 |
| 140 |  | 1500 | 90000 | 20 | 520 | 801.00 | 8 | 300 | 0 | 0 | 0 | 0 | 21.4 | 84.74 |
| 141 |  | 1500 | 90000 | 20 | 520 | 801.00 | 8 | 300 | 0 | 0 | 0 | 0 | 22.1 | 78.03 |
| 142 |  | 1500 | 90000 | 20 | 520 | 801.00 | 8 | 300 | 0 | 0 | 0 | 0 | 19.65 | 77.62 |
| 143 |  | 1500 | 90000 | 20 | 520 | 801.00 | 8 | 300 | 0 | 0 | 0 | 0 | 20.85 | 67.08 |
| 144 |  | 1500 | 90000 | 20 | 520 | 801.00 | 8 | 300 | 0 | 0 | 0 | 0 | 19.2 | 76.67 |
| 145 |  | 1500 | 90000 | 20 | 520 | 801.00 | 8 | 300 | 0 | 0 | 0 | 0 | 20.89 | 81.65 |
| 146 |  | 1500 | 90000 | 20 | 520 | 801.00 | 8 | 300 | 0 | 0 | 0 | 0 | 23.38 | 59.71 |
| 147 |  | 1500 | 90000 | 20 | 520 | 801.00 | 8 | 300 | 0 | 0 | 0 | 0 | 17.3 | 79.72 |
| 148 |  | 1500 | 90000 | 20 | 520 | 801.00 | 8 | 300 | 0 | 0 | 0 | 0 | 23.36 | 57.98 |
| 149 |  | 1500 | 90000 | 20 | 520 | 801.00 | 8 | 300 | 0 | 0 | 0 | 0 | 17.67 | 88.53 |
| 150 |  | 1500 | 90000 | 20 | 520 | 801.00 | 8 | 300 | 0 | 0 | 0 | 0 | 20.83 | 80.21 |
| 151 |  | 1500 | 90000 | 20 | 520 | 801.00 | 8 | 300 | 0 | 0 | 0 | 0 | 17.06 | 85.52 |
| 152 |  | 1500 | 90000 | 20 | 520 | 801.00 | 8 | 300 | 0 | 0 | 0 | 0 | 15.85 | 89.7 |
| 153 |  | 1500 | 90000 | 20 | 520 | 801.00 | 8 | 300 | 0 | 0 | 0 | 0 | 19.04 | 84.5 |
| 154 |  | 1500 | 90000 | 20 | 520 | 801.00 | 8 | 300 | 0 | 0 | 0 | 0 | 16.44 | 81.7 |
| 155 |  | 1500 | 90000 | 20 | 520 | 801.00 | 8 | 300 | 0 | 0 | 0 | 0 | 14.76 | 95.24 |
| 156 |  | 1500 | 90000 | 20 | 520 | 801.00 | 8 | 300 | 0 | 0 | 0 | 0 | 15.97 | 78.2 |
| 157 |  | 1500 | 90000 | 20 | 520 | 801.00 | 8 | 300 | 0 | 0 | 0 | 0 | 17.06 | 88.82 |
| 158 | [19] | 900 | 39900 | 36.66 | 372 | 921.69 | 6 | 90 | 0 | 0 | 0 | 0 | 0.0735 | 263.2921 |
| 159 |  | 900 | 39900 | 36.66 | 372 | 921.69 | 6 | 90 | 0 | 0 | 0 | 0 | 0.124 | 263.2921 |
| 160 |  | 900 | 39900 | 36.66 | 372 | 921.69 | 6 | 90 | 0 | 0 | 0 | 0 | 0.2053 | 263.2921 |
| 161 |  | 900 | 39900 | 36.66 | 372 | 921.69 | 6 | 90 | 0 | 0 | 0 | 0 | 0.265 | 263.2921 |
| 162 |  | 550 | 76500 | 39.2 | 362.8 | 2050.20 | 10 | 80 | 0 | 0 | 0 | 0 | 0.04 | 599.76 |
| 163 |  | 550 | 76500 | 39.2 | 362.8 | 2050.20 | 10 | 80 | 0 | 0 | 0 | 0 | 0.08 | 599.76 |
| 164 |  | 550 | 76500 | 39.2 | 362.8 | 2050.20 | 10 | 80 | 0 | 0 | 0 | 0 | 0.16 | 599.76 |
| 165 |  | 1250 | 58750 | 25.22 | 429 | 581.63 | 6 | 82 | 0 | 0 | 0 | 0 | 0.1129 | 370.4188 |
| 166 |  | 1000 | 36000 | 24.57 | 415.6 | 831.60 | 6 | 80 | 0 | 0 | 0 | 0 | 0.145 | 238.8204 |
| 167 |  | 1000 | 36000 | 24.57 | 415.6 | 831.60 | 6 | 80 | 0 | 0 | 0 | 0 | 0.16 | 238.8204 |
| 168 |  | 1000 | 36000 | 24.57 | 415.6 | 831.60 | 6 | 80 | 0 | 0 | 0 | 0 | 0.18 | 238.8204 |
| 169 |  | 1000 | 36000 | 24.57 | 415.6 | 831.60 | 6 | 80 | 0 | 0 | 0 | 0 | 0.24 | 238.8204 |
| 170 |  | 1000 | 36000 | 24.57 | 415.6 | 831.60 | 6 | 80 | 0 | 0 | 0 | 0 | 0.05 | 238.8204 |
| 171 |  | 1000 | 36000 | 24.57 | 415.6 | 831.60 | 6 | 80 | 0 | 0 | 0 | 0 | 0.19 | 238.8204 |
| 172 |  | 455 | 81000 | 34.29 | 431.9 | 1692.90 | 8 | 150 | 0 | 0 | 0 | 0 | 0.1835 | 555.498 |
| 173 |  | 455 | 81000 | 34.29 | 431.9 | 1692.90 | 8 | 150 | 0 | 0 | 0 | 0 | 0.186 | 555.498 |
| 174 |  | 455 | 81000 | 34.29 | 431.9 | 1692.90 | 8 | 150 | 0 | 0 | 0 | 0 | 0.2045 | 555.498 |
| 175 |  | 390 | 81000 | 34.29 | 431.9 | 1692.90 | 8 | 150 | 0 | 0 | 0 | 0 | 0.129 | 555.498 |
| 176 |  | 520 | 81000 | 34.29 | 431.9 | 1692.90 | 8 | 150 | 0 | 0 | 0 | 0 | 0.211 | 555.498 |
| 177 |  | 1200 | 79500 | 39.66 | 400 | 1661.55 | 8 | 100 | 0 | 0 | 0 | 0 | 0.0576 | 315.297 |
| 178 |  | 1200 | 79500 | 39.66 | 400 | 1661.55 | 8 | 100 | 0 | 0 | 0 | 0 | 0.082 | 945.891 |
| 179 |  | 1200 | 79500 | 39.66 | 400 | 1661.55 | 8 | 100 | 0 | 0 | 0 | 0 | 0.178 | 945.891 |
| 180 |  | 2300 | 135000 | 33.89 | 362 | 2173.50 | 8 | 92.5 | 0 | 0 | 0 | 0 | 0.081 | 457.515 |
| 181 |  | 2300 | 135000 | 33.89 | 362 | 2173.50 | 8 | 92.5 | 0 | 0 | 0 | 0 | 0.5385 | 457.515 |
| 182 |  | 2300 | 135000 | 33.89 | 362 | 2173.50 | 8 | 92.5 | 0 | 0 | 0 | 0 | 0.227 | 457.515 |
| 183 |  | 1200 | 54000 | 25 | 460 | 691.20 | 6 | 100 | 0 | 0 | 0 | 0 | 0.09 | 253.8 |
| 184 |  | 1200 | 54000 | 25 | 460 | 691.20 | 6 | 100 | 0 | 0 | 0 | 0 | 0.13 | 253.8 |
| 185 |  | 1200 | 54000 | 25 | 460 | 691.20 | 6 | 100 | 0 | 0 | 0 | 0 | 0.16 | 253.8 |
| 186 |  | 1200 | 54000 | 25 | 460 | 691.20 | 6 | 100 | 0 | 0 | 0 | 0 | 0.22 | 253.8 |
| 187 |  | 650 | 36000 | 25 | 403.7 | 831.60 | 6 | 100 | 0 | 0 | 0 | 0 | 0.24 | 169.2 |
| 188 |  | 650 | 36000 | 22.04 | 403.7 | 831.60 | 6 | 90 | 0 | 0 | 0 | 0 | 0.041 | 182.4912 |
| 189 |  | 650 | 36000 | 22.04 | 403.7 | 831.60 | 6 | 90 | 0 | 0 | 0 | 0 | 0.062 | 182.4912 |
| 190 |  | 650 | 36000 | 22.04 | 403.7 | 831.60 | 6 | 90 | 0 | 0 | 0 | 0 | 0.09 | 182.4912 |
| 191 |  | 650 | 36000 | 22.04 | 403.7 | 831.60 | 6 | 90 | 0 | 0 | 0 | 0 | 0.114 | 182.4912 |
| 192 |  | 650 | 36000 | 22.04 | 403.7 | 831.60 | 6 | 90 | 0 | 0 | 0 | 0 | 0.142 | 182.4912 |
| 193 |  | 650 | 36000 | 22.04 | 403.7 | 831.60 | 6 | 90 | 0 | 0 | 0 | 0 | 0.168 | 182.4912 |
| 194 |  | 650 | 36000 | 22.04 | 403.7 | 831.60 | 6 | 90 | 0 | 0 | 0 | 0 | 0.198 | 182.4912 |
| 195 |  | 650 | 34000 | 35.39 | 384.77 | 523.60 | 6 | 100 | 0 | 0 | 0 | 0 | 0.168 | 156.4238 |
| 196 |  | 650 | 36000 | 22.04 | 359 | 831.60 | 6 | 100 | 0 | 0 | 0 | 0 | 0.0152 | 238.032 |
| 197 |  | 650 | 36000 | 22.04 | 359 | 831.60 | 6 | 100 | 0 | 0 | 0 | 0 | 0.0218 | 238.032 |
| 198 |  | 650 | 36000 | 22.04 | 359 | 831.60 | 6 | 100 | 0 | 0 | 0 | 0 | 0.0283 | 238.032 |
| 199 |  | 650 | 36000 | 22.04 | 359 | 831.60 | 6 | 100 | 0 | 0 | 0 | 0 | 0.0155 | 396.72 |
| 200 |  | 650 | 36000 | 22.04 | 359 | 831.60 | 6 | 100 | 0 | 0 | 0 | 0 | 0.0216 | 396.72 |
| 201 |  | 650 | 36000 | 22.04 | 359 | 831.60 | 6 | 100 | 0 | 0 | 0 | 0 | 0.0285 | 396.72 |
| 202 |  | 1000 | 38000 | 19.43 | 373 | 1527.60 | 8 | 60 | 0 | 0 | 0 | 0 | 0.0476 | 295.336 |
| 203 |  | 1000 | 38000 | 19.43 | 373 | 1527.60 | 8 | 60 | 0 | 0 | 0 | 0 | 0.0332 | 295.336 |
| 204 |  | 1000 | 38000 | 19.43 | 373 | 1527.60 | 8 | 60 | 0 | 0 | 0 | 0 | 0.079 | 295.336 |
| 205 |  | 1000 | 84000 | 33.34 | 525 | 1150.80 | 6 | 100 | 0 | 0 | 0 | 0 | 0.0473 | 1120.224 |
| 206 |  | 1000 | 84000 | 33.34 | 525 | 1150.80 | 6 | 100 | 0 | 0 | 0 | 0 | 0.0713 | 1120.224 |
| 207 |  | 1000 | 84000 | 33.34 | 525 | 1150.80 | 6 | 100 | 0 | 0 | 0 | 0 | 0.0939 | 1120.224 |
| 208 |  | 1000 | 84000 | 33.34 | 525 | 1150.80 | 6 | 100 | 0 | 0 | 0 | 0 | 0.0555 | 1120.224 |
| 209 |  | 1000 | 84000 | 33.34 | 525 | 1150.80 | 6 | 100 | 0 | 0 | 0 | 0 | 0.0287 | 1120.224 |
| 210 |  | 1000 | 84000 | 33.34 | 525 | 1150.80 | 6 | 100 | 0 | 0 | 0 | 0 | 0.1349 | 1120.224 |
| 211 |  | 1000 | 84000 | 33.34 | 525 | 1150.80 | 6 | 100 | 0 | 0 | 0 | 0 | 0.1673 | 1120.224 |
| 212 |  | 1100 | 82500 | 39.66 | 441 | 1724.25 | 8 | 100 | 0 | 0 | 0 | 0 | 0.0663 | 327.195 |
| 213 |  | 1100 | 82500 | 39.66 | 441 | 1724.25 | 8 | 100 | 0 | 0 | 0 | 0 | 0.185 | 327.195 |
| 214 |  | 1100 | 82500 | 39.66 | 441 | 1724.25 | 8 | 100 | 0 | 0 | 0 | 0 | 0.0679 | 981.585 |
| 215 |  | 1100 | 82500 | 39.66 | 441 | 1724.25 | 8 | 100 | 0 | 0 | 0 | 0 | 0.163 | 981.585 |
| 216 |  | 1650 | 108500 | 30.65 | 411.25 | 3515.40 | 8 | 70 | 0 | 0 | 0 | 0 | 0.106 | 332.5525 |
| 217 |  | 1650 | 108500 | 35.08 | 411.25 | 3515.40 | 8 | 70 | 0 | 0 | 0 | 0 | 0.111 | 761.236 |
| 218 |  | 1650 | 108500 | 29.6 | 411.25 | 3515.40 | 8 | 70 | 0 | 0 | 0 | 0 | 0.228 | 321.16 |
| 219 |  | 1650 | 108500 | 30.58 | 411.25 | 3515.40 | 8 | 70 | 0 | 0 | 0 | 0 | 0.098 | 663.586 |
| 220 |  | 800 | 148000 | 37.41 | 382 | 2101.60 | 8 | 60 | 0 | 0 | 0 | 0 | 0.0077 | 1107.336 |
| 221 |  | 800 | 148000 | 37.41 | 382 | 2101.60 | 8 | 60 | 0 | 0 | 0 | 0 | 0.0141 | 1107.336 |
| 222 |  | 800 | 148000 | 37.41 | 382 | 2101.60 | 8 | 60 | 0 | 0 | 0 | 0 | 0.0176 | 1107.336 |
| 223 |  | 800 | 148000 | 41.24 | 382 | 2101.60 | 8 | 60 | 0 | 0 | 0 | 0 | 0.0067 | 1220.704 |
| 224 |  | 800 | 148000 | 41.24 | 382 | 2101.60 | 8 | 60 | 0 | 0 | 0 | 0 | 0.0064 | 1220.704 |
| 225 |  | 800 | 148000 | 41.24 | 382 | 2101.60 | 8 | 60 | 0 | 0 | 0 | 0 | 0.0089 | 1220.704 |
| 226 |  | 800 | 148000 | 28.23 | 382 | 2101.60 | 8 | 60 | 0 | 0 | 0 | 0 | 0.0273 | 835.608 |
| 227 |  | 800 | 148000 | 28.23 | 382 | 2101.60 | 8 | 60 | 0 | 0 | 0 | 0 | 0.0456 | 835.608 |
| 228 |  | 800 | 148000 | 28.23 | 382 | 2101.60 | 8 | 60 | 0 | 0 | 0 | 0 | 0.0572 | 835.608 |
| 229 |  | 375 | 58250 | 31.37 | 408.8 | 879.58 | 4 | 60 | 0 | 0 | 0 | 0 | 0.035 | 365.4605 |
| 230 |  | 375 | 58250 | 29.97 | 408.8 | 879.58 | 4 | 60 | 0 | 0 | 0 | 0 | 0.063 | 349.1505 |
| 231 |  | 375 | 58250 | 32.53 | 408.8 | 879.58 | 4 | 60 | 0 | 0 | 0 | 0 | 0.114 | 378.9745 |
| 232 |  | 500 | 56250 | 34.47 | 389 | 849.38 | 4 | 30 | 0 | 0 | 0 | 0 | 0.042 | 387.7875 |
| 233 |  | 500 | 56250 | 34.47 | 389 | 849.38 | 4 | 30 | 0 | 0 | 0 | 0 | 0.043 | 387.7875 |
| 234 | [20] | 1100 | 40000 | 18.42 | 414 | 924.00 | 6 | 80 | 0 | 0 | 0 | 0 | 4 | 163.5 |
| 235 |  | 1100 | 40000 | 9.6 | 386 | 924.00 | 6 | 80 | 0 | 0 | 0 | 0 | 18 | 222.36 |
| 236 |  | 1260 | 60000 | 18.15 | 452 | 618.00 | 8 | 100 | 0 | 0 | 0 | 0 | 9 | 117.72 |
| 237 |  | 1260 | 60000 | 10.61 | 520 | 618.00 | 8 | 300 | 0 | 0 | 0 | 0 | 20 | 143.8 |
| 238 | [21] | 1000 | 62500 | 37.5 | 420 | 256.25 | 6 | 6 | 0 | 0 | 0 | 0 | 9.5 | 1339 |
| 239 |  | 1000 | 62500 | 37.5 | 420 | 256.25 | 6 | 6 | 0 | 0 | 0 | 0 | 13.3 | 1320 |
| 240 | [22] | 750 | 14400 | 27.93 | 333.2 | 313.92 | 6 | 65 | 0 | 0 | 0 | 0 | 3 | 425.5 |
| 241 |  | 750 | 14400 | 27.93 | 333.2 | 313.92 | 6 | 65 | 0 | 0 | 0 | 0 | 7 | 257 |
| 242 |  | 750 | 14400 | 27.93 | 333.2 | 313.92 | 6 | 65 | 0 | 0 | 0 | 0 | 6 | 215 |
| 243 |  | 750 | 14400 | 27.93 | 333.2 | 313.92 | 6 | 65 | 0 | 0 | 0 | 0 | 4 | 320 |
| 244 |  | 750 | 14400 | 27.93 | 333.2 | 313.92 | 6 | 65 | 0 | 0 | 0 | 0 | 7.5 | 310 |
| 245 |  | 750 | 14400 | 27.93 | 333.2 | 313.92 | 6 | 65 | 0 | 0 | 0 | 0 | 17 | 235 |
| 246 |  | 750 | 14400 | 27.93 | 333.2 | 313.92 | 6 | 65 | 0 | 0 | 0 | 0 | 3.5 | 460 |
| 247 |  | 750 | 14400 | 27.93 | 333.2 | 313.92 | 6 | 65 | 0 | 0 | 0 | 0 | 5 | 440 |
| 248 |  | 750 | 14400 | 27.93 | 333.2 | 313.92 | 6 | 65 | 0 | 0 | 0 | 0 | 10 | 245 |

**Table S2. Details of the collected dataset.**

| **Parameters** | **Symbol** | **Unit** |
| --- | --- | --- |
| Height of column | $H$ | mm |
| Gross area of column | $A_{g}$ | mm^2^ |
| Compressive strength of concrete | $f_{c}^{'}$ | MPa |
| Yield strength of longitudinal steel | $f_{y}$ | MPa |
| Area of longitudinal steel | $A_{s}$ | mm^2^ |
| Diameter of transverse steel | $D_{t}$ | mm |
| Stirrup spacing | $S_{v}$ | mm |
| Modulus of Elasticity of FRP | $E_{f}$ | GPa |
| Tensile strength of FRP | $f_{t}$ | MPa |
| Nos. of FRP layers | *n* | − |
| FRP Thickness | $t_{f}$ | mm |
| Type of Fabric | $F_{t}$ | − |
| Corrosion Percentage | *η* | % |
| Axial strength of the column | $P_{u}$ | kN |

**Fig. S1.** Distribution of input and output parameters (a) *H*, (b) *A_g_*, (c) *f^’^_c_*, (d) *f_y_*, (e) *A_s_*, (f) *D_t_*, (g) *S_v_*, (h) *E_f_*, (i) *f_t_*, (j) *n*t_f_*, (k) *F_t_*, (l) *η* and (m) *P_u_*.

**References**

[1] Radhi, M.S., Hassan, M.S. & Gorgis, I.N. Carbon fibre-reinforced polymer confinement of corroded circular concrete columns. *J. Build. Eng.* **43**, 102611, <https://doi.org/10.1016/j.jobe.2021.102611> (2021).

[2] Tastani, S.P. & Pantazopoulou, S.J. Experimental evaluation of FRP jackets in upgrading RC corroded columns with substandard detailing. *Eng. Struct.* **26**, 817-829, <https://doi.org/10.1016/j.engstruct.2004.02.003> (2004).

[3] Mohammed, A.A. *et al*. Behavior of damaged concrete columns repaired with novel FRP jacket. *J. Compos. Constr*. **23**, 04019013, <https://doi.org/10.1061/(ASCE)CC.1943-5614.0000942> (2019).

[4] Lee, C. *et al.* Accelerated corrosion and repair of reinforced concrete columns using carbon fibre reinforced polymer sheets. *Can. J. Civ. Eng.* **27**, 941-948, <https://doi.org/10.1139/l00-030> (2000).

[5] Bae, S.W. and Belarbi, A. Effects of corrosion of steel reinforcement on RC columns wrapped with FRP sheets. *J. Perform. Constr. Facil*. **23**, 20-31, <https://doi.org/10.1061/(ASCE)0887-3828(2009)23:1(20)> (2009).

[6] Kashi, A., Ramezanianpour, A.A. & Moodi, F. Durability evaluation of retrofitted corroded reinforced concrete columns with FRP sheets in marine environmental conditions. *Constr. Build. Mater*. **151**, 520-533, <https://doi.org/10.1016/j.conbuildmat.2017.06.137> (2017).

[7] Chenchen, L.I., Aimin, Y.U., Danying, G.A.O. & Pu, Z.H.A.N.G. Experimental study on axial compression of corroded reinforced concrete columns strengthened with FRP strips under erosion environment. *J. Compos. Mater*. **37**, 2015-2028, <https://doi.org/10.13801/j.cnki.fhclxb.20200212.005> (2020).

[8] Pantazopoulou, S.J., Bonacci, J.F., Sheikh, S., Thomas, M.D.A. & Hearn, N. Repair of corrosion-damaged columns with FRP wraps. *J. Compos. Constr*. **5**, 3-11, <https://doi.org/10.1061/(ASCE)1090-0268(2001)5:1(3)> (2001).

[9] Belarbi, A. & Bae, S.W. An experimental study on the effect of environmental exposures and corrosion on RC columns with FRP composite jackets. *Compos. B. Eng*. **38**, 674-684, <https://doi.org/10.1016/j.compositesb.2006.09.004> (2007).

[10] Xiang, Z., Wang, J., Niu, J., Zhou, J. & Wang, J. Axial compressive responses of concrete canvas and CFRP reinforced corroded concrete short columns. *Case Stud. Constr. Mater.* **17**, 01661, <https://doi.org/10.1016/j.cscm.2022.e01661> (2022).

[11] Neale, K.W., Demers, M. & Labossiere, P. FRP protection and rehabilitation of corrosion-damaged reinforced concrete columns. *Int. J. Mater*. **23**, 348-371, <https://doi.org/10.1504/IJMPT.2005.007735> (2005).

[12] Zhou, Y.W., Wu, L.Y., Sui, L.L. & Xing, F. Experimental studies on the mechanical performances of corroded reinforced concrete columns retrofitted with FRP. *Appl. Mech. Mater.* **405**, 726-730, <https://doi.org/10.4028/www.scientific.net/AMM.405-408.726> (2013).

[13] Ma, Y., Che, Y. & Gong, J. Behavior of corrosion damaged circular reinforced concrete columns under cyclic loading. *Constr. Build. Mater*. **29**, 548-556, <https://doi.org/10.1016/j.conbuildmat.2011.11.002> (2012).

[14] Zhou, H. *et al.* Partially corroded reinforced concrete piers under axial compression and cyclic loading: An experimental study. *Eng. Struct.* **203**, 109880, <https://doi.org/10.1016/j.engstruct.2019.109880> (2020).

[15] Radhi, M., Hassan, M.S. and Gorgis, I.N., Compressive performance of corroded reinforced concrete columns. *Eng. Technol*. **38**, 1618-1628, <https://doi.org/10.30684/etj.v38i11A.1545> (2020).

[16] Yuan, Z., Fang, C., Parsaeimaram, M. & Yang, S. Cyclic behavior of corroded reinforced concrete bridge piers. *J. Bridge Eng*. **22**, 04017020, <https://doi.org/10.1061/(ASCE)BE.1943-5592.0001043> (2017).

[17] Wu, X., Chen, L., Li, H. & Xu, J. Experimental study of the mechanical properties of reinforced concrete compression members under the combined action of sustained load and corrosion. *Constr. Build. Mater.* **202**, 11-22, <https://doi.org/10.1016/j.conbuildmat.2018.12.156> (2019).

[18] Meda, A., Mostosi, S., Rinaldi, Z. & Riva, P. Experimental evaluation of the corrosion influence on the cyclic behaviour of RC columns. *Eng. Struct.* **76**, 112-123, <http://dx.doi.org/10.1016/j.engstruct.2014.06.043> (2014).

[19] Dai, K.Y., Yu, X.H. & Lu, D.G. Phenomenological hysteretic model for corroded RC columns. *Eng. Struct*. **210**, 110315, <https://doi.org/10.1016/j.engstruct.2020.110315> (2020).

[20] Vu, N.S., Yu, B. and Li, B. Prediction of strength and drift capacity of corroded reinforced concrete columns. *Constr. Build. Mater*. **115**, 304-318, <https://doi.org/10.1016/j.conbuildmat.2016.04.048> (2016).

[21] Al-Akhras, N. & Al-Mashraqi, M. Repair of corroded self-compacted reinforced concrete columns loaded eccentrically using carbon fiber reinforced polymer. *Case Stud. Constr. Mater.* **14**, 00476, <https://doi.org/10.1016/j.cscm.2020.e00476> (2021).

[22] Li, Q., Dong, Z., He, Q., Fu, C. and Jin, X. Effects of reinforcement corrosion and sustained load on mechanical behavior of reinforced concrete columns. *Materials* **15**, 3590, <https://doi.org/10.3390/ma15103590> (2022).
